# Building Better Barrels – β-barrel Biogenesis and Insertion in Bacteria and Mitochondria

**DOI:** 10.1016/j.jmb.2021.166894

**Published:** 2021-02-24

**Authors:** Kathryn A. Diederichs, Susan K. Buchanan, Istvan Botos

**Affiliations:** Laboratory of Molecular Biology, National Institute of Diabetes & Digestive & Kidney Diseases, National Institutes of Health, 9000 Rockville Pike, Bethesda, MD 20892, USA

**Keywords:** outer membrane beta-barrels, BAM complex, TOM complex, SAM complex, Oep80

## Abstract

β-barrel proteins are folded and inserted into outer membranes by multi-subunit protein complexes that are conserved across different types of outer membranes. In Gram-negative bacteria this complex is the barrel-assembly machinery (BAM), in mitochondria it is the sorting and assembly machinery (SAM) complex, and in chloroplasts it is the outer envelope protein Oep80. Mitochondrial β-barrel precursor proteins are translocated from the cytoplasm to the intermembrane space by the translocase of the outer membrane (TOM) complex, and stabilized by molecular chaperones before interaction with the assembly machinery. Outer membrane bacterial BamA interacts with four periplasmic accessory proteins, whereas mitochondrial Sam50 interacts with two cytoplasmic accessory proteins. Despite these major architectural differences between BAM and SAM complexes, their core proteins, BamA and Sam50, seem to function the same way. Based on the new SAM complex structures, we propose that the mitochondrial β-barrel folding mechanism follows the budding model with barrel-switching aiding in the release of new barrels. We also built a new molecular model for Tom22 interacting with Sam37 to identify regions that could mediate TOM-SAM supercomplex formation.

## Introduction

**Figure 1.**
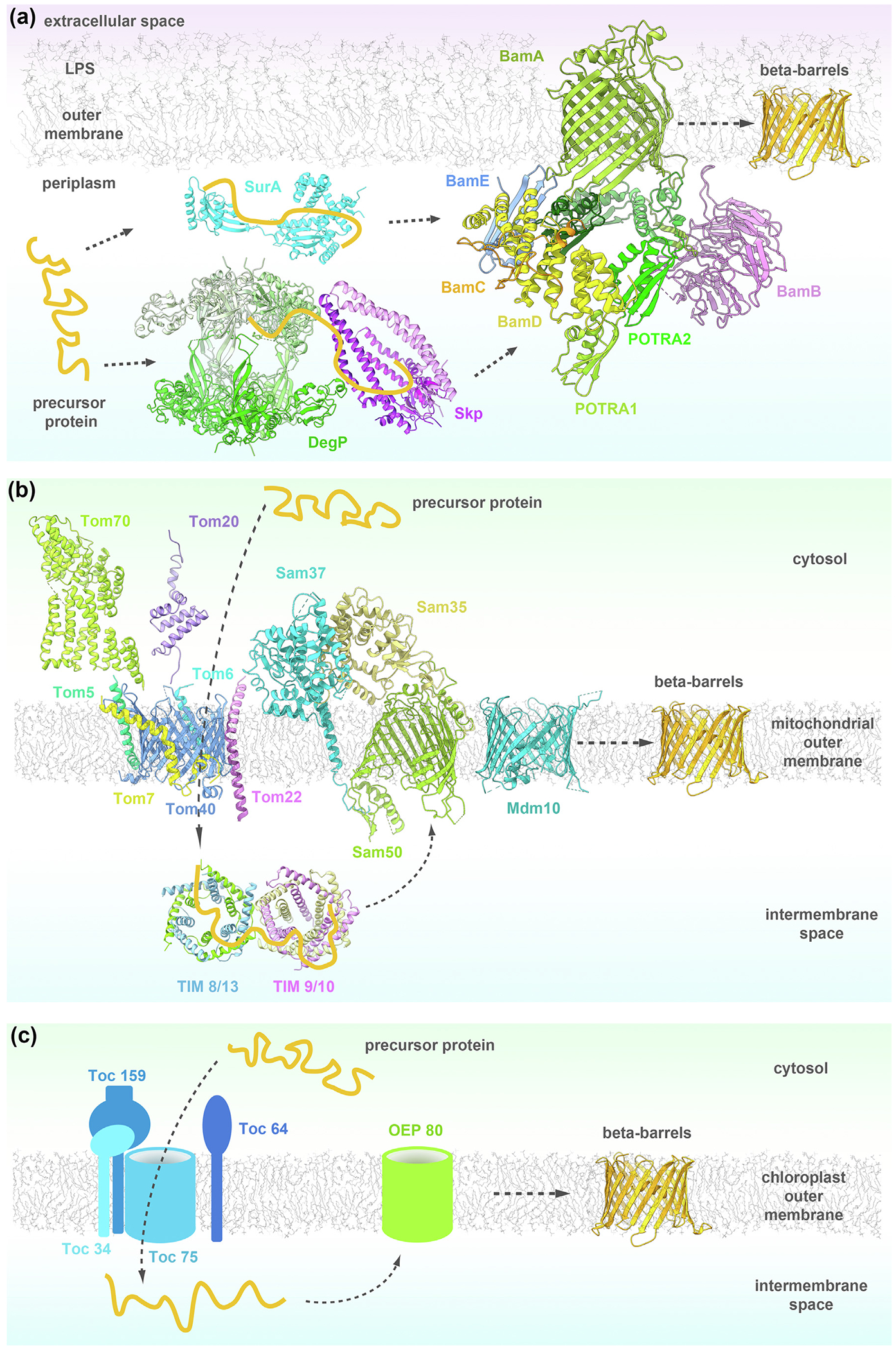
β-barrel biogenesis machineries in bacteria, mitochondria and chloroplasts. (a) In bacteria the precursor proteins (orange) translocated to the periplasm are kept from aggregation by the chaperones SurA (cyan; PDB: 1M5Y), Skp (violet; 1SG2) and DegP (green; 1KY9) and passed on to the BAM complex (5D0O), formed by BamA (green), BamB (orchid), BamC (orange), BamD (yellow) and BamE (light blue). The BAM complex folds and inserts the new β-barrel (orange) into the outer membrane. (b) In mitochondria the barrel precursor proteins are translocated to the IMS by the TOM complex (6UCU), composed of Tom40 (blue), Tom22 (orchid), Tom5 (green), Tom6 (light blue), Tom7 (yellow), Tom20 (purple; 1OM2) and Tom70 (light green; 2GW1). The translocated precursors are kept from aggregation by the chaperones TIM8/13 (blue/green; 3CJH) and TIM9/10 (khaki/orchid; 3DXR) and transferred to the SAM complex (6WUT) formed by Sam50 (light green), Sam35 (tan) and Sam37 (light blue). The SAM complex folds and inserts the new β-barrel (orange) into the mitochondrial outer membrane (MOM). Mdm10 (turquoise; 7BTX) assists the SAM complex. (c) In chloroplasts the precursor proteins are translocated into the IMS by the TOC complex, composed of Toc75, Toc34, Toc159 and Toc64. From the TOC complex they are transferred to Oep80 which folds and integrates the new β-barrel (orange) into the chloroplast outer membrane.

A double layer of membranes protects Gram-negative bacteria, chloroplasts and mitochondria: an outer and an inner membrane. β-barrel proteins are found exclusively in outer membranes of bacteria and organelles with an endosymbiotic origin, such as mitochondria and plastids.^1–4^ These integral membrane proteins are formed by sheets of β-strands wrapped into a barrel where the first strand hydrogen-bonds the last strand. β-barrel proteins can be structural proteins, enzymes, and can form pores that passively or actively transport metabolites across the membrane or can be involved in protein translocation across the membrane and protein insertion into the membrane.^1^ Folding and insertion of these β-barrels into the outer membrane is carried out by multi-subunit protein complexes that are conserved across different types of outer membranes. In Gram-negative bacteria, this is the barrel-assembly machinery (BAM) complex (Figure 1 (a)).^5,6^ Its equivalent in mitochondria is the sorting and assembly machinery (SAM) complex (Figure 1 (b)), ^7–9^ whereas in chloroplasts it was suggested that the outer envelope protein Oep80 fulfills this function (Figure 1 (c)).^10^ While we know that these complexes are involved in β-barrel biogenesis, the exact mechanism of folding and insertion of β-barrels into the outer membrane is still not fully understood.

## Bacterial machineries

**Figure 2.**
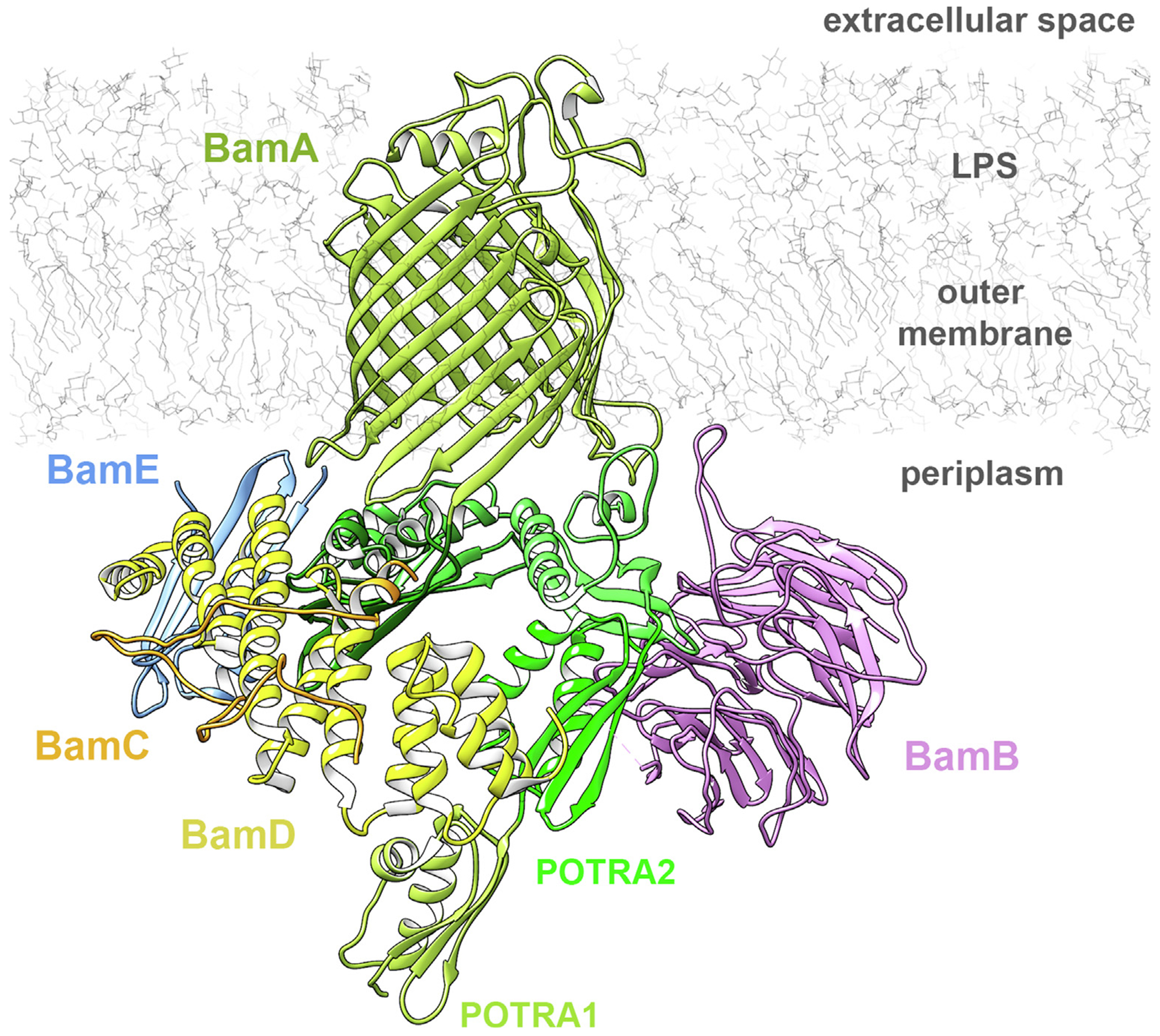
Structure of the BAM complex (PDB: 5D0O). The core component BamA (light green) is a bacterial outer membrane β-barrel with five periplasmic POTRA domains (different shades of green). The lipoproteins BamB (orchid), BamC (orange), BamD (yellow) and BamE (light blue) bind to the periplasmic side of BamA.

Gram-negative bacteria protect themselves from the harsh extracellular environment by a cell envelope built from an inner membrane, a periplasmic space and an outer membrane. The outer membrane is populated by outer membrane proteins (OMPs) which are mostly β-barrel proteins embedded in the membrane. The β-barrels can be differentiated by their number of strands, oligomerization or function but they share a common property: they are folded and inserted into the membrane by the same barrel-assembly machinery (BAM) complex. Typically, the BAM complex consists of an integral membrane protein, BamA, and four lipoproteins: BamB, C, D and E (Figure 2). However, the composition of BAM can vary between bacteria.^5,11,12^ The *Neisseria* BAM complex lacks BamB,^13^ while *Nostoc, Thermosynechococcus elongatus, Rhodothermus marinus* have fewer Bam lipoproteins or none. BamA seems to have acquired more lipoprotein subunits through evolution.^14^ The Bam lipoproteins are anchored on the periplasmic side of the outer membrane. The structures of all individual components have been determined over the past decade and recently structures of full BAM complexes were also reported.^15–20^

## BamA (also known as Omp85, D15, YaeT)

BamA is an essential 88 kDa protein that forms the core of the BAM complex and is conserved across all Gram-negative bacteria.^21,22^ It is part of the Omp85 superfamily of outer membrane proteins, which are 16-strand β-barrel proteins implicated in protein secretion and membrane protein insertion in bacteria and organelles.^23,24^ The C-terminal transmembrane β-barrel is linked to soluble, periplasmic polypeptide transport-associated (POTRA) domains.^21,25^ While *Escherichia coli* BamA has five POTRA domains, *Myxococcus xanthus* can have up to seven.^26^ The POTRA domains have a well-conserved βααββ fold and mediate interactions with the four Bam lipoproteins.^27^ It was also suggested that they assist in OMP folding by β-augmentation of unfolded barrels with their β-strands.^27,28^ The most C-terminal POTRA is the best conserved across species, followed by the most N-terminal one.^26^ The number of POTRA domains required for cell viability vary between species, though most require at least the C-terminal POTRA. POTRA 3–5 are essential in *E. coli* while *Neisseria meningitidis* only requires POTRA5 for survival.^27,29^

The BamA β-barrel consists of 16 anti-parallel β-strands where the first and last strands hydrogen-bond to close the barrel. In *Haemophilus ducreyi* BamA (*Hd*BamA), there are 8H-bonds, whereas in *Neisseria gonorrhoeae* (*Ng*BamA) there are only 2, leading to two different conformations of the lateral gate region (Supplementary Figure 1). The destabilization of the C-terminal β-strand may facilitate access from the lumen of the barrel to the lipid phase of the membrane through this lateral gate. While the lumen of the barrel is almost empty, the extracellular loops form a dome above the barrel, isolating the inside from the extracellular space.^30^

## BamB (also known as YfgL)

BamB is not a core component of the BAM complex but the lack of this component compromises the viability of cells.^31,32^ It is a 40 kDa lipoprotein with an eight-bladed β-propeller fold homologous to eukaryotic WD40 repeat domains.^33,34^ It was suggested that BamB acts as a scaffold for the BamA POTRA domains and other Bam lipoproteins as well as chaperones involved in the folding. BamB interacts with BamA on the hinge region between the POTRA domains 2 and 3.^35^

## BamC (also known as NlpB)

BamC is a 34 kDa lipoprotein for which mainly structures of its fragments are available. The N-terminal domain is very flexible and was solved only in complex with BamD, while the two C-terminal helix-grip domains are stable.^36,37^ The function of BamC alone remains unknown as does its role in the BamCD complex. In three of the full BAM complex structures (5D0O, 5AYW, 5EKQ; Table 1) both the N-terminal domain and the first helix-grip domain interact with BamD, contradicting the surface-exposure observations.^38,39^ It is possible that exposure to detergents during the purification process disrupts the native conformation of BamC.^40^ The N-terminal part of BamC is more defined in the crystal structure of the BAM complex in cymal + octylglucoside (6LYS, Table 1) where it interacts with BamA POTRA1.^19^

## BamD (also known as YfiO)

BamD is also a core component of the BAM complex. It is a 26 kDa lipoprotein, essential for bacterial survival.^5^ The structure contains five tetratricopeptide repeat (TPR) domains and binds directly to POTRA5 of BamA.^41,42^ Some studies show β-signal recognition of unfolded OMPs by BamD, but this specificity was not proven for all BAM substrates.^43^ Some studies suggest that BamD may activate BamA, but the mechanism of this process is still unknown.^44^

## BamE (also known as SmpA)

BamE is an 11 kDa lipoprotein with an ααβββ fold that is not essential for cell viability. It interacts directly with BamD and forms an interface between BamD and BamA.^45,15–17^

## The BAM complex

It was shown that the functional BAM complex is monomeric and each Bam protein contributes a single copy to the assembled 200 kDa complex.^5,6,46,47^ Several fully assembled BAM complex structures were reported by X-ray crystallography and cryo-EM.^15–20^ Eight structures have all Bam components, while two of the structures lack BamB (5D0Q, 5EKQ; Table 1). The Bam lipoproteins interact with BamA along the base of the β-barrel and the POTRA domains (Figure 2). BamB interacts with the hinge region between POTRA2 and POTRA3, whereas BamCDE interact mainly with POTRA5, parts of POTRA4 and the β-barrel periplasmic loops. The BAM complex structures show the POTRA domain in either an open or closed state. In the closed state, the POTRA domain is at the base of the barrel, blocking the barrel lumen from periplasmic access, while in the open state, the displaced POTRA domain permits barrel access. The BamA barrel was also observed in two conformations: ‘inward-open’ or ‘outward-open’. As in other outer membrane proteins, the base of the barrel is fully open to the periplasm in the ‘inward-open’ state. This state can undergo a conformational twist by constricting the periplasmic side of the barrel and slightly opening the top of the barrel to yield the ‘outward-open’ state. The ‘outward-open’ state seems to coordinate with the closed POTRA state. However, it is still unclear what role these states play in OMP biogenesis.^40^ It is important to note that the currently available BAM structures are either in detergent or a lipid nanodisc environment, and both have a different effect on the structure. While cryo-EM structures in DDM are all open, the 4.2 Å structure in nanodisc is in a closed conformation.^19^ Therefore, the structure of the BAM complex in a native lipid environment, perhaps obtained using SMALPs,^48^ might represent the most unperturbed state of the machinery.

Bacterial chaperones play an important role in stabilizing the barrel precursor proteins after they emerge from the inner membrane Sec translocon and are delivered to the BAM complex. The precursor protein translocated to the periplasm has its signal peptide cleaved off by the signal peptidase^49^ then is bound by either the SurA or Skp/DegP chaperones (Supplementary Resource 1).

## Mitochondrial machineries

A major distinguishing feature of eukaryotes is the presence of a nucleus, and the evolution of this organelle required a better energy source that could only be provided by mitochondria.^50^ During a key evolutionary step of eukaryotes, a bacterial endosymbiont was acquired by its ancestor and adapted into the mitochondrion.^51,52^ Mitochondria contain more than 1000 different proteins, most of which are imported from the cytosol.^53^

Organelle β-barrel proteins are evolutionarily related to bacterial outer membrane β-barrel proteins.^54^ However, while in bacteria the outer membrane borders the extracellular space, the mitochondrial outer membrane (MOM) borders the cytoplasm. Bacterial OMPs are synthesized in the cytoplasm and transported to the periplasm through the Sec translocation machinery, whereas mitochondrial OMPs synthesized in the cytoplasm have to be first translocated across the same MOM where they will be inserted (Figure 1(b)). The MOM protein sorting and assembly is realized through three machineries: the translocase of the outer membrane (TOM) complex, the sorting and assembly machinery (SAM) complex and the mitochondrial distribution and morphology (MDM) complex.^55^ The transfer of translocated proteins from TOM to SAM is assisted by the small translocase of the inner membrane (TIM) chaperones.

**Figure 3.**
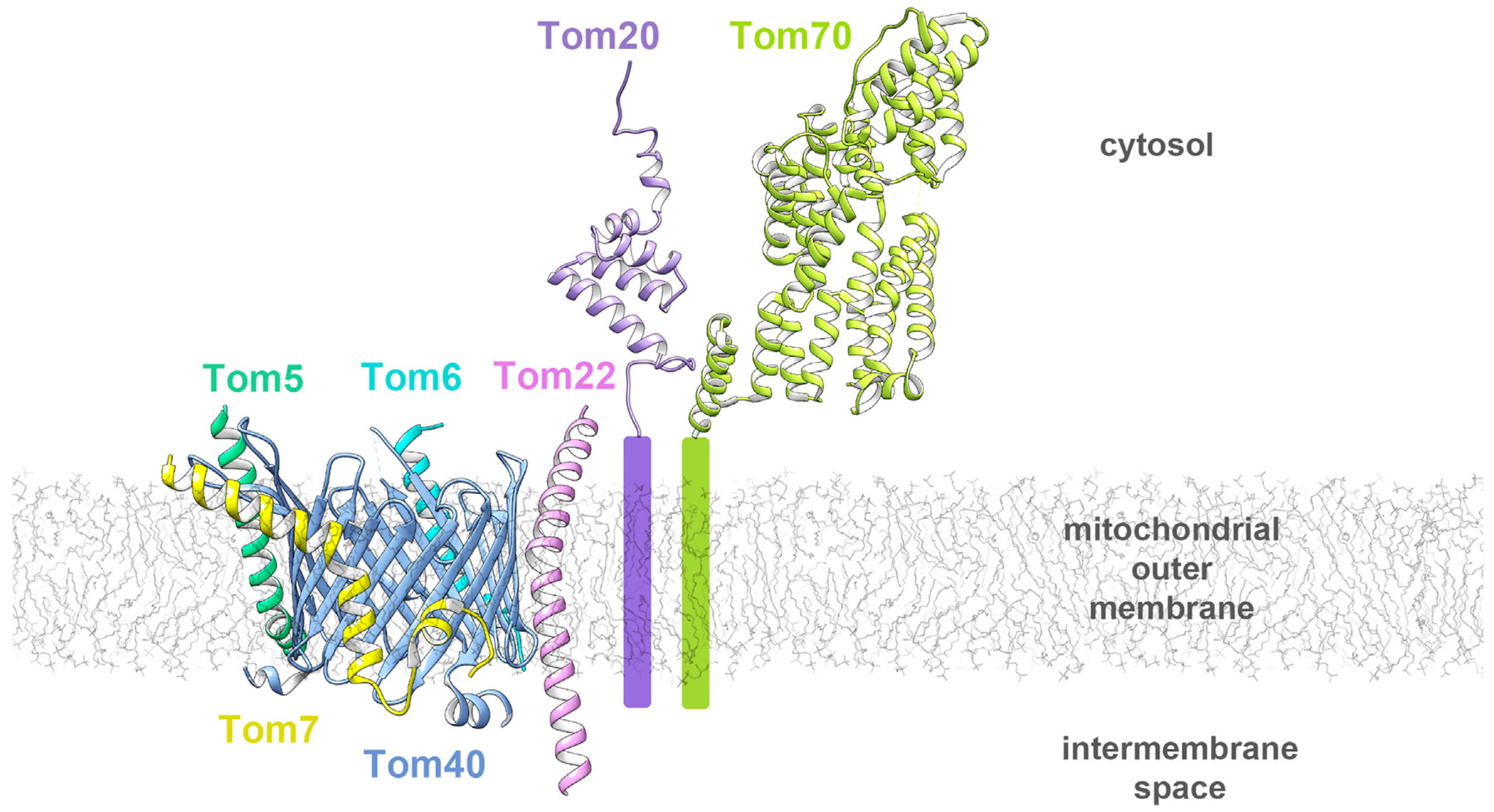
Structure of the TOM complex. A monomeric TOM core complex (PDB: 6UCU) is composed of Tom40, a MOM β-barrel (blue), and several peripheral proteins anchored in the membrane by a single α-helix: Tom22 (orchid), Tom5 (green), Tom6 (light blue) and Tom7 (yellow). In addition to the core, the receptor proteins Tom20 (purple; 1OM2) and Tom70 (light green; 2GW1) are also part of the complex.

The TOM complex in the MOM is the entry gate for most mitochondrial proteins.^56–59^ It consists of seven transmembrane proteins: Tom40, Tom22, Tom5-7, Tom70 and Tom20.^60,61^ The first five proteins form a very stable core complex, while Tom70 and Tom20 readily dissociate from the core complex in the presence of detergent.^62^ The NMR structure of the Tom20 cytosolic part first became available,^63^ followed by the X-ray structure of the cytosolic part of Tom70,^64^ a medium-resolution cryo-EM structure of the *Neurospora crassa* TOM core complex,^65^ and atomic resolution cryo-EM structures of the *Saccharomyces*^66,67^ and human TOM core complexes^68^ (Figure 3). The central component of the TOM complex is the β-barrel Tom40 through which pre-proteins enter, while the six other proteins are anchored by transmembrane α-helices.^69–71^ However, not all proteins go through the Tom40 barrel. Alpha-helical proteins are thought to be recognized by Tom70 then passed to the MIM complex for membrane insertion.^72^ The discussion here is limited to fungal TOM complexes as these have been the major model systems for past functional analyses, as well as more recent structural analyses.

## Tom40

The translocation pore in the TOM complex is a 40 kDa integral β-barrel protein that is structurally related to the major mitochondrial porin, the voltage-dependent anion-selective channel (VDAC).^73^ Tom40 and VDAC belong to the ‘eukaryotic porins’ superfamily of β-barrel proteins with no direct bacterial predecessor.^74^ The β-barrel contains 19 antiparallel β-strands with the exception of strands β1 and β19, which are parallel.^66,67^ On the mitochondrial inter-membrane space (IMS) side of the barrel, there are two N-terminal α-helical segments of which α1 lies flat on the membrane surface and α2 spans the interior of the barrel. The barrel and the N-terminal segment have the same structural features as VDAC despite only a ~15% sequence identity.^75^ The C-terminal end of β19 from the barrel continues with a third α3 helix that follows with an unstructured tail pointing from the IMS into the barrel. This C-terminal tail may act as an autoinhibitory element that is displaced from the pore by a precursor protein. In a dimer, two Tom40 barrels directly interact through hydrophobic sidechains in β1-β19-β18 but tilt away by ~40°.^66^ The interior surface of the barrel is highly negatively charged, a feature that might allow it to favorably bind positively charged presequences to initiate translocation. The pore could still vertically fit 1–2 helices and is unlikely to laterally open to the membrane since β1 and β19 are sealed by ten H-bonds.^66^

## Tom5, Tom6 and Tom7

These small α-helical transmembrane proteins help with the TOM complex stability and assembly (Figure 3). They tightly interact with the outside of the Tom40 β-barrel and contain several proline residues that kink their α-helices. In the *Saccharomyces* structures, Tom5 has a kink and interacts with β9–β11 of Tom40, while the N-terminal hydrophilic part of its helix is oriented toward the cytosol. The IMS portion of the Tom5 helix interacts with α1 of Tom40. The Tom6 transmembrane helix has a kink and interacts with β13-β15 of Tom40 through 3 pairs of conserved residues. Tom7 has a noticeably kinked transmembrane helix and interacts with β1-β6 of Tom40 through two pairs of conserved residues.^67^

## Tom22 (also known as Mas22)

Tom22 is a secondary receptor protein with a 45 residue longbow-shaped helix, the middle of which spans the membrane. The kink is caused by a conserved Pro112 residue, which is important for mitochondrial targeting of Tom22 and stability of the TOM complex.^76^ The helix follows the contour of the Tom40 barrel, with α-β contacts mainly mediated by twelve pairs of conserved hydrophobic residues on β15-β18 of Tom40.^67^ The Tom22 helix extends into the IMS and may provide a binding site for presequences or for the TIM complex.^77,78^ On the cytoplasmic side, the amphipathic helix lies flat on the membrane surface and a cluster of acidic residues might be responsible for presequence binding. Cytoplasmic residues 1–88 are not visible in the structures but are involved in Tom20 and Tom70 binding.^79,80^ Two Tom22 transmembrane helices are wedged into the interface between two Tom40 β-barrels.^66^ Tom9 from plant mitochondria is a Tom22 ortholog with a smaller, basic cytosolic domain, that cannot bind presequences but can still interact with Tom20.^81,82^

## Tom20 (also known as Mas20)

Tom20 is the small transmembrane receptor protein in the TOM complex that recognizes preprotein N-terminal mitochondrial targeting sequences. The N terminus is anchored in the membrane, whereas the cytosolic C terminus contains a Glu-rich region and a single TPR motif with an apolar groove that can bind a presequence helix mainly through hydrophobic interactions.^63^ The positively charged region of the presequence is a distinct recognition element from the region that binds Tom20. Photo-crosslinking studies show that a hydrophobic groove on α-helix 1 can interact with the cytoplasmic region of Tom22, that can compete with presequence binding to the groove.^80^ The plant Tom20 ortholog is C-terminally anchored in the membrane and contains two TPR motifs.^83^ These orthologs share conserved residues if their sequences are aligned in an antiparallel way, and while their function is analogous their evolutionary origin is likely different.^84^ Crosslinking studies identified Tom20 association with Tom40 cytosolic loops,^76^ however it remains unclear how the Tom20 transmembrane helix associates with the core TOM complex.

## Tom70

Tom70 is the large receptor protein in the TOM complex that binds internal targeting sequences of preproteins while it also has a co-chaperone function. It is formed by eleven TPR motifs organized into a right-handed superhelix. The N terminus of the protein is anchored in the outer mitochondrial membrane by a transmembrane helix and the cytosolic N- and C-terminal domains are connected through a disordered linker region.^64^ The N-terminal domain with TPR motifs 1–3 has a peptide-binding groove for Hsp70 and Hsp90, while the C-terminal domain forms a conserved pocket for binding presequence peptides. The presequence can bind to the open conformation binding cleft, created by shifting the N-terminal domain away from the C-terminal domain.^85^ Only a portion of the total mitochondrial Tom70 is associated with the TOM complex, Tom70 being enriched at the sites of contact between the outer and inner membranes.^60^ Plants harbor the homologous mitochondrial OM64 that is N-terminally anchored in the membrane but not associated with the TOM complex and is a paralog of Toc64.^86^ The structural basis for Tom70 association with the core complex is currently unknown, specifically where the Tom70 TM helix interacts. The cytosolic domain of Tom70 has been shown to interact with the cytosolic domain of Tom22.^79^ Deletion of Tom6 reduces the amount of Tom70 co-precipitated with Tom40,^87^ suggesting that Tom6 may mediate the interaction of Tom70 and Tom40 in the membrane.

## The TOM complex

The oligomeric state of the TOM complex is still highly debated. Recent structures show either a dimeric or tetrameric complex, but trimeric assemblies were observed by early EM and crosslinking studies.^76^ The Tom40 barrel is tightly surrounded by the TM helices of the Tom proteins. The dimerization occurs at the β1-β19-β18 region of Tom40 and also wedges two Tom22 helices into the interface. Upon rotation of Tom22 and shift of Tom7 the dimer may transition into a trimer. Tom20 might also facilitate trimerization. The tetrameric complex has a non-symmetrical structure, a dimer of the TOM core complex dimer.^66^ It is possible that TOM complexes multimerize to increase the efficiency of protein translocation.^66^

On the cytoplasmic side of the mitochondrial membrane, helical presequences of proteins are recognized by Tom70 and Tom20, then the positively charged helical regions interact with the Tom40 barrel for translocation. After passing through the Tom40 barrel, the preproteins bind the small TIM hexameric complexes which guide the precursors to the TIM22 complex in the inner mitochondrial membrane or the SAM complex in the outer mitochondrial membrane.

The TPR motifs present in Tom20, Tom70, and BamC seem to be conserved presequence binding motifs for membrane translocation complexes both in bacteria and eukaryotes. TPR motifs may represent ancient protein-protein interaction modules adapted by chaperones, transcription proteins, cell-cycle and protein transport complexes for their specific functions.^88^

While significant progress was made in understanding the structural intricacies of the TOM machinery, the full structures of the individual TOM proteins are still not known. This includes Tom20 and Tom70 for which only the soluble portion of the structure was determined and the topological arrangement of these elements relative to the full TOM complex is not clear. Regions of TOM proteins important for interactions with other complexes, like the SAM complex, are not structurally known. The details of TOM-SAM supercomplex formation cannot be fully understood in the absence of structural details. It is also unclear if Tom20 and Tom70 are associated with the TOM-SAM supercomplex, and if so where they bind. The mechanism of β-barrel precursor transfer from the TOM complex to the SAM complex, as well as the folding state of the precursors during this process, are also unknown.

The precursor proteins translocated by the TOM complex need to maintain their folding state in the IMS while they reach their destinations in the inner or outer membrane. IMS chaperones of the translocase of the inner membrane (TIM) play a crucial role in maintaining aggregation-prone polypeptides in their elongated form. Transition of β-barrel precursors from the TOM to either the SAM or TIM22 complexes is assisted by the small TIM chaperones TIM8/13 and TIM9/10 (Figure 1 (b), Supplementary Resource 2)^89,90^

**Figure 4.**
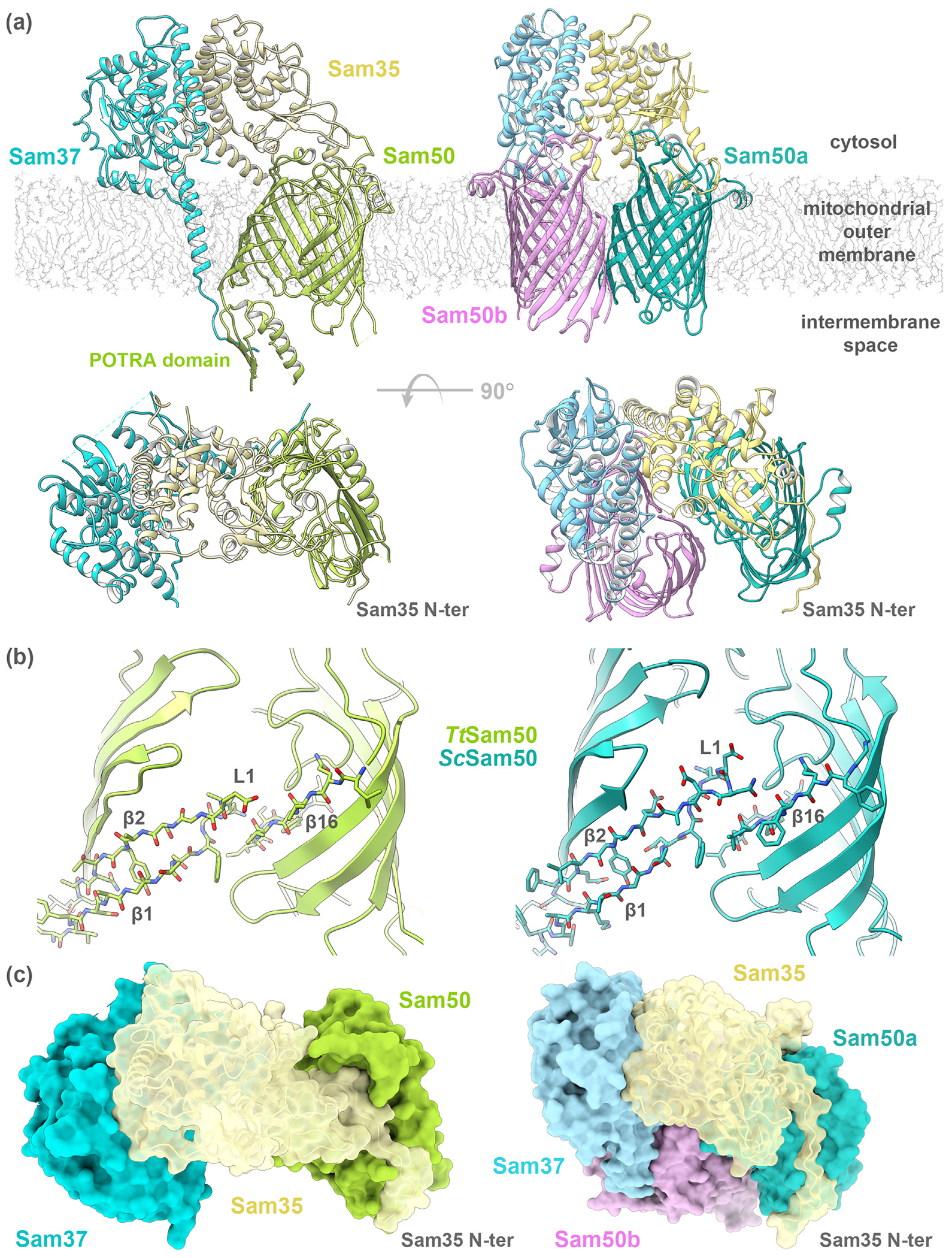
Structures of the SAM complex (PDB: 6WUT, 7BTW). (a) Two views of the complex formed by Sam50, a MOM β-barrel (green), Sam35 (tan) and Sam37 (light blue). *Tt*SAM complex on the left, *Sc*SAM monomer + Sam50b on the right with Sam50b in orchid. (b) The Sam50 barrel lateral gate formed by β1 and β16 strands has no H-bonds in *Tt*Sam50 or *Sc*Sam50. (c) SAM complex molecular surface shows the cytosolic side of the Sam50 barrel occluded by the Sam35 N terminus. *Tt*SAM complex on the left, *Sc*SAM monomer + Sam50b on the right.

The sorting and assembly machinery (SAM) complex (also called topogenesis of mitochondrial outer membrane β-barrel proteins (TOB) complex) is located in the outer mitochondrial membrane and facilitates the folding and insertion of β-barrel proteins into the outer membrane. The SAM complex is composed of three subunits: Sam50, Sam35, and Sam37. Sam50 is the membrane-spanning β-barrel core of the complex while the two smaller subunits, Sam35 and Sam37, associate on the cytosolic side of the membrane (Figure 4(a)).^8^,^9^,^99^,^91–98^ The first high resolution structures of the SAM complex from the fungi *Thermothelomyces thermophilus* (*Tt*SAM) and *Saccharomyces cerevisiae* (*Sc*SAM) were recently determined using cryo-EM (Table 1)^96,99^ These structures confirm the SAM complex topology proposed by biochemical experiments. The β-barrel precursor proteins processed by the SAM complex contain a conserved sequence motif on the most C-terminal β-strand, called the β-signal.^100^

## Sam50 (also known as Tob55)

Sam50 is the 50 kDa β-barrel core of the SAM complex and is evolutionarily related to BamA from Gram-negative bacteria.^8^ Prior to the recent high resolution SAM complex structures,^96,99^ most biochemical characterization was completed using Sam50 homology models based on BamA. The SAM complex structures revealed that Sam50 is indeed a 16-stranded β-barrel with one POTRA domain extending into the IMS (Figure 4(a)).^96^ Sam50 specifically binds the precursor protein β-signal and remains associated with the growing β-barrel until it is completely folded.^24,100^

Knockdown or depletion of Sam50 results in reduced levels of SAM complex accessory proteins, as well as reduced import and assembly of MOM β-barrel proteins.^95,101,102^ The β-barrel domain of Sam50 is essential for cell viability and β-barrel biogenesis,^7,92,100–102^ while the POTRA domain is not.^100,103,104^ Sam50 POTRA deletion constructs exhibit normal growth phenotypes and association with accessory subunits for SAM complex formation.^96,100,104^ While Sam50 POTRA deletion constructs bind β-signal and β-barrel precursor proteins like wildtype,^24,100,103^ the precursor proteins remain associated with the SAM complex suggesting that the POTRA domain is involved in precursor release from the complex.^100,104^

The β-barrel of Sam50 contains a lateral gate, formed by β1 and β16. Crosslinking studies of *Sc*Sam50 demonstrated that β1 and β16 interact in the absence of β-barrel precursor protein’ and that the precursor β-signal displaces β16 to specifically interact with β1.^24^ In both the *Tt*Sam50 and *Sc*Sam50 structures (6WUT, 7BTW; Table 1), no hydrogen bonds are present between β1 and β16 to close the Sam50 lateral gate (Figure 4(b)), suggesting flexibility and the capability to accommodate a β-barrel precursor protein.^96,99^

The Sam50 cytosolic loops fold over the top of the barrel, occlude access from the cytosol and prevent precursor protein efflux out of the barrel. The T/Sam50 IMS loops and POTRA domain are oriented to allow access to the barrel lumen’ while the *Sc*Sam50 POTRA domain density is not resolved. Cytosolic loop 6 contains the (V/I)RG(F/Y) motif conserved across the Omp85 family^21^ (Supplementary Figure 2), and interacts with the β-barrel interior to stabilize the barrel.^96^ Loop 6 is essential for yeast cell viability, and the (V/I)RG(F/Y) motif is required for Sam50 β-signal binding and β-barrel precursor protein interaction with the SAM complex.^24^

## Sam35 (also known as Tob38, Tom38)

Sam35 is also essential for cell viability and β-barrel biogenesis.^95,97,105,106^ In higher eukaryotes, Metaxin 2 fulfills the role of Sam35.^107^ Both Sam35 and Metaxin 2 are peripheral membrane proteins that interact with Sam50 and Sam37 (Metaxin 1) on the cytosolic side of the membrane.^94,95,97,100,107^ Upon depletion of Sam50, mitochondrial levels of Sam35 and Sam37 are reduced’ further demonstrating a requirement of Sam50 for Sam35 mitochondrial localization.^95,101^

The SAM complex structures revealed that Sam35 is anchored to the membrane through many interactions with Sam50 and Sam37.^96,99^

The N terminus of Sam35 interacts with the cytosolic loops of Sam50 (Figure 4(c)), which is stabilized by Sam35 interactions with the cytosolic domain of Sam37 (Figure 4(a)). Deletion of the Sam35 N terminus results in reduced association with Sam50 but does not change interaction with Sam37.^96^ Sam35 has a GST-like fold, but does not contain active site residues required for GST activity.^96^

Pull-down assays demonstrated that Sam35 specifically binds precursor β-signal in the presence and absence of Sam50.^100^ In fact, the presence of Sam35 is required for Sam50 to bind β-barrel precursor proteins.^93,100^

## Sam37 (also known as Tob37, Mas37, Tom37)

Unlike the other subunits in the SAM complex, Sam37 is not essential for growth or β-barrel assembly at permissive temperatures.^9,91^ In higher eukaryotes, Metaxin 1 fills the functional role of Sam37.^101,106,107^ Sam37 is exposed on the cytosolic side of the membrane,^9,91,105,106^ and is predicted to have between zero to two C-terminal TM helices depending on the species (Supplementary Figure 3, Supplementary Table 1).^91^,^96^,^105–107^ Metaxin 1 is predicted to have one C-terminal transmembrane domain, which is important for mitochondrial targeting and membrane association.^106,107^ The C-terminal sequence conservation for Sam37 is very low, further supporting the variation in number of predicted TM helices (Supplementary Figure 3).

The *Tt*SAM complex structure contains density for one TM helix, however two TM helices are predicted based on TMHMM 2.0 analysis.^108^ The *Tt*Sam37 TM helix does not interact with the cytosolic loops or β-barrel of *Tt*Sam50. The linker region between the *Tt*Sam37 TM helix and the second predicted TM helix does interact with the *Tt*Sam50 POTRA domain in the IMS.^96^ Similar to Sam35, Sam37 also contains a GST-like fold which is missing key active site residues necessary for GST activity.

Sam37 interacts with the cytosolic domains of two different MOM proteins, Sam35 and Tom22. The cytosolic domain of Sam37 functions to localize Sam35 to the MOM^107^ and stabilize the Sam35 interaction with Sam50.^93,100^ This function is supported by the extensive interactions between Sam37 and Sam35 observed in the available structures.^96,99^ Sam37 also mediates TOM-SAM supercomplex formation via interaction with Tom22.^109^

## The SAM complex (also known as TOB complex)

The SAM complex is composed of three subunits (Sam50, Sam35, Sam37), each contributing one copy to form the core complex (also referred to as the SAM monomer) (Figure 4(a)). The SAM complex primarily functions to fold and insert β-barrel proteins into the MOM, however some data suggests that it also facilitates biogenesis of α-helical TOM complex proteins.^110,111^ β-barrel precursor proteins are recognized by the SAM complex through specific interactions of the β-signal with Sam35 and Sam50.^24,100^ Single channel electrophysiology recordings demonstrated that purified SAM complex but not Sam50 alone forms a channel sensitive to β-signal, indicating that either Sam35 or Sam37 contribute to β-signal sensitivity.^100^ Therefore, it is unclear if either Sam35 or Sam50 serves as the receptor protein, or how these two essential subunits coordinate β-barrel precursor protein recognition.

The overall arrangement of the subunits is similar between the *Tt*SAM and *Sc*SAM complex structures. The Sam50 β-barrel cytosolic loops are capped by the Sam35 N terminus. Sam37 does not contact Sam50 on the cytosolic side of the membrane but interacts extensively with Sam35 in both structures.

The key difference between the structures is the presence of an additional β-barrel in the *Sc*SAM complex structures. *Sc*Sam37 caps either another copy of Sam50 (SAM monomer + Sam50b structure; PDB 7BTW) or Mdm10 (SAM monomer + Mdm10 structure; 7BTX, 7BTY). It is proposed that the additional β-barrel serves as a placeholder until the folding precursor protein displaces it.^99^ The second β-barrel is not present in the *Tt*SAM complex structures, however *Tt*SAM complex dimers (two copies each of Sam50, Sam37, and Sam35) in a non-physiological up-down orientation were observed in detergent (Supplementary Resource 4). Some of the dimer populations contained Sam50 subunits with β1- β4 rotated outwards, suggesting flexibility to accommodate folding precursor protein (Supplementary Movie 1).

*Tt*Sam37 contains one transmembrane helix and interacts with *Tt*Sam50 POTRA in the IMS,^96^ which may facilitate precursor release from the complex.^93,96,100,104^ Since Sam37 does not contain predicted TM helices in all species, precursor release from the complex must be facilitated independent of POTRA domain interaction with Sam37 in those species.

Sam37 associates with Sam35 in the cytosol through many interactions. Sequence conservation mapped to the structure of Sam37 does not suggest any functional clues as the highly conserved regions are not localized,^96^ nor are residues that interact with Sam35 or Sam50 (Supplementary Figure 3). Nonetheless, the sheer number of interactions supports the Sam37 functional role of stabilizing Sam35.^93,100^

The N terminus of Sam35 interacts with the cytosolic loops of Sam50, together occluding access to the Sam50 β-barrel from the cytosol and preventing precursor protein efflux out of the barrel lumen.^96,99^ Sam35 sequence alignments identify a fair number of highly conserved residues (Supplementary Figure 4), however these residues are not localized to any particular part of the structure. There is one groove of semi-conserved residues on *Tt*Sam35 that could be a potential binding site, although this has yet to be experimentally tested.

Sequence conservation of Sam50 is the highest in the C-terminal region with loop 6 and the β-signal. Within the highly conserved β16, which contains the β-signal, an absolutely conserved glycine residue forms a kink in the β-strand that likely aids the lateral gate opening of Sam50.^112^ It is interesting to note that the other side of the lateral gate, β1, is not as highly conserved despite its specific binding of precursor β-signal in the crosslinking studies.^24^

The fungal mitochondrial outer membrane contains four types of β-barrel proteins (Sam50, Tom40, VDAC/Porin, and Mdm10), all of which are processed by the SAM complex.^113–115^ The rates of β-barrel biogenesis differ between the types of β-barrel protein, with Tom40 being the slowest to be released from the complex.^8,9,111,116^ It has been suggested that the slow release of Tom40 allows the other TOM complex subunits to associate.^72^ The SAM complex forms a supercomplex with another outer membrane β-barrel, Mdm10, to facilitate Tom22 biogenesis and promote release of Tom40 from the SAM complex.^117–119^

While the structures are in agreement with the biochemical data, the molecular mechanisms of β-barrel precursor protein recognition, folding, and insertion by the SAM complex have yet to be determined. Of particular interest is how Sam35 is involved in precursor protein recognition from the cytosolic side of the membrane, since the precursor is in the mitochondrial IMS. Additionally, whether Sam37 and the Sam50 POTRA domain coordinate precursor protein release, and how this process differs in species where Sam37 does not contain transmembrane domains, is unknown. Lastly, the specific regions of the SAM complex required for outer membrane supercomplex formation with the TOM complex have yet to be identified.

## Molecular model of the TOM-SAM supercomplex

**Figure 5.**
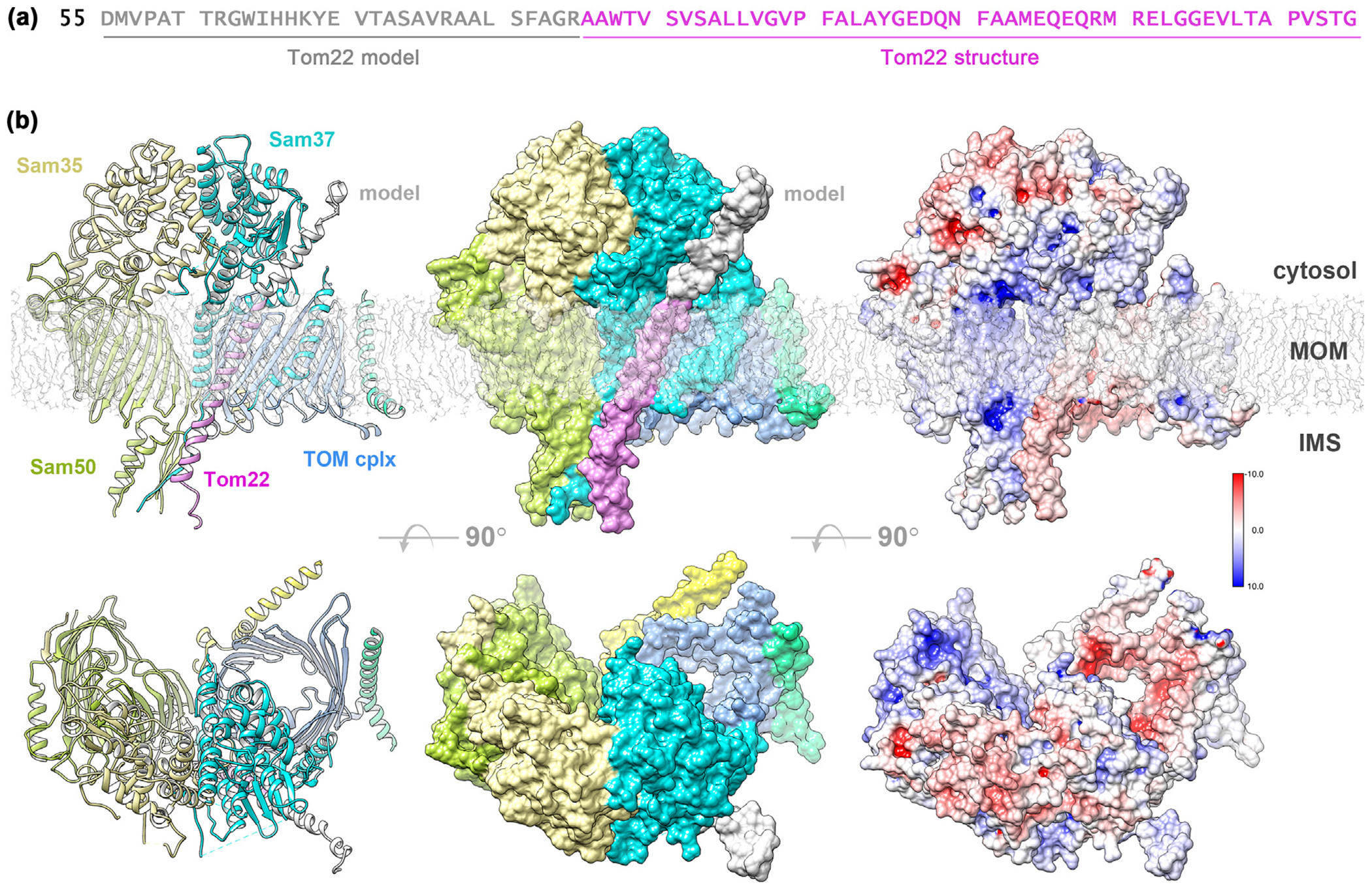
Model of the TOM-SAM supercomplex. (a) *Tt*Tom22 sequence (UniProt G2QBG3) used in the model with the *ab-initio* modeled part (grey) and the homologous *Sc*Tom22 structure (orchid). (b) Cartoon representation, molecular surface and surface charges for the TOM-SAM supercomplex viewed from the membrane plane and from the cytosol *Tt*Tom22 cartoon is shown in orchid while the *Tt*Tom22 molecular surface shows the TM helix (orchid) homologous to the *Sc*Tom22 structure (PDB: 6UCU, 6JNF) and the modeled part (grey). This *Tt*Sam37-*Tt*Tom22 interaction accommodates the rest of the SAM and TOM complexes without clashes while maintaining their proper orientation in the mitochondrial membrane. *Tt*Tom22 model was built by DMPfold^179^ on the PSIPRED server^180^ and used for protein–protein interaction prediction with the standalone version of PIPER.^181^ Surface charges calculated in Chimera using the AMBER forcefield and APBS.^182^

Previous studies have demonstrated that TOM-SAM supercomplex formation is mediated by a cytosolic interaction between Tom22 and Sam37.^109,120^ Pull-down assays with Tom22 truncation mutants found that the Tom22 N-terminal 54 residues are not involved in this interaction. Therefore, we modeled the *T. thermophilus* Tom22-Sam37 interaction with a truncated Tom22 sequence (residues 55–135) (Figure 5(a)). While the structure of the *Sc*Tom22 transmembrane helix is known,^66,67^ the cytosolic N terminus of *Tt*Tom22 (residues 55–85) was modeled *ab-initio*. The modeled *Tt*Tom22 contains a positively charged cytosolic domain with limited rotational freedom and an extended TM α-helix that fits in the context of the known ScTOM core complex.^66,67^ Of the two negatively charged regions on the surface of *Tt*Sam37, one was identified as the interaction surface of *Tt*-Sam37-*Tt*Tom22 by rigid body docking. The TOM-SAM supercomplex was generated by superimposing the remaining subunits onto the interacting *Tt*-Sam37-*Tt*Tom22 complex (Figure 5(b)). The supercomplex maintains proper topology in the mitochondrial membrane. The TOM and SAM complex subunits fit into the supercomplex model without major clashes and without position adjustments which further supports our model. It should be noted that Tom20 and Tom70 were not taken into account in the supercomplex model, as neither are part of the TOM core complex.

Considering the low sequence conservation of the Sam37 C terminus and the variation in TM helices between species (Supplementary Figure 3), it is likely that the N terminus of Sam37 is involved in the Tom22-Sam37 interaction as this region is more conserved. This is consistent with our model and further validates it. The cytosolic domain of Tom22 is not resolved in the reported TOM complex structures^66,67^ suggesting increased flexibility in this region. The *Tt*Tom22 interaction with *Tt*Sam37 in our model is a result of rigid-body docking, therefore conformational changes of the *Tt*Tom22 N terminus could further improve this interaction. Since conformational changes are not taken into account in our model, we cannot characterize it in atomic detail. *Tt*Sam37 residues 35–63 and 326–421 are involved in the *Tt*Tom22 interaction and residues 38–48 are conserved.^96^

In the TOM-SAM supercomplex model, the Tom40 and Sam50 β-barrels sit adjacent to one another. The association with the SAM complex results in the displacement of one of the TOM complexes from the TOM homodimer. The TOM complex is thought to form homodimers and homotrimers to increase its stability,^76^ therefore the replacement of one TOM complex by the SAM complex could be energetically favorable.

## Mdm10

In fungi, two additional β-barrel proteins Mdm10 and Mmm2 (Mdm34) are important for mitochondrial dynamics and morphology (Figure 1b).^121^ Mdm10 might be another component of the SAM complex, since deletion of Mdm10 gives rise to abnormal mitochondrial morphology.^122^ Mdm10 might exist in a complex with Mmm1 and Mdm12, involved in β-barrel biogenesis^123^ and is also part of the endoplasmic reticulum-mitochondria encounter structure (ERMES) complex that tethers the ER to mitochondria.^124^ However, the SAM-mediated protein assembly and ER-mitochondria contact are two different functions of Mdm10, realized by residues on opposite sides of its β-barrel.^118^ When bound to the SAM complex, Mdm10 is involved in the release of β-barrel preproteins from the complex.^117^ The SAM-Mdm10 complex is also involved in the assembly of the α-helical Tom22 with Tom40.^122^ The structure of *S. cerevisiae* Mdm10 (*Sc*Mdm10) interacting with Sam50 of the *Sc*SAM complex was recently solved (7BTY, 7BTX; Table 1),^99^ however the interacting residues do not include those previously identified biochemically.^118^ Phylogenetically, Mdm10 belongs to the VDAC/Tom40 ‘eukaryotic porin’ superfamily, which is supported by the 19-stranded β-barrel observed in the structure. However, Mdm10 is unlike VDAC since it exposes large loops to both sides of the membrane that do not seem to be essential for its function.^119^ These large loops were not visible in the cryo-EM density, likely due to high flexibility.^99^

## Chloroplast machineries

Chloroplasts or plastids evolved by the incorporation of a cyanobacterium into an ancestral eukaryotic cell.^125^ About 95% of the chloroplast proteins are encoded in the nucleus and have to be imported into the chloroplasts.^126^ Preproteins require an N-terminal chloroplast transit peptide (cTP) as a targeting signal, with a highly heterogeneous sequence but overall positive charge.^127^ The lack of consensus for chloroplast targeting is striking and contrasts the conserved mitochondrial targeting signal. While the mechanism of β-barrel-biogenesis is not well understood in chloroplasts, the equivalent machinery involved in the process includes the translocase of the outer membrane of chloroplast (TOC) and Oep80 proteins (Figure 1(c)). The TOC complex includes Toc75, Toc34 and Toc159 (Supplementary Resource 3).^128^

## Comparison of the BAM and SAM complexes

β-barrel biogenesis in Gram-negative bacteria, mitochondria, and chloroplasts is facilitated by evolutionary related protein complexes (BAM, SAM, and TOC complexes, respectively). The β-barrel core of each complex is well conserved; BamA in Gram-negative bacteria, Sam50 in mitochondria, and Oep80 in chloroplasts (Supplementary Figure 2)^8,129^ All form (or are assumed to form in the case of Oep80) a 16-stranded β-barrel with highly conserved cytosolic/extracellular loop 6 and β16. Additionally, this β-barrel is oriented in the membrane so that the POTRA domain(s) point toward the IMS (mitochondria and chloroplasts) or periplasm (Gram-negative bacteria),^8,27,130^ where the precursor proteins approach from. Since the structure of Oep80 has yet to be solved, this discussion will focus on comparison of the BAM and SAM complexes.

The SAM and BAM complexes are each composed of multiple subunits, all associated with the conserved β-barrel core (Sam50 or BamA).^73,129^ While Sam50 and BamA share sequence homology and the same topology, the accessory subunits for each complex are unique. There is no sequence homology between the accessory proteins (Sam35, Sam37, and BamB-E), nor similarities in interaction with the β-barrel core. Furthermore, the BAM accessory subunits are located on the opposite side of the outer membrane from the SAM accessory subunits (Figure 1 (a) and (b)), suggesting distinct roles in each system.

**Figure 6.**
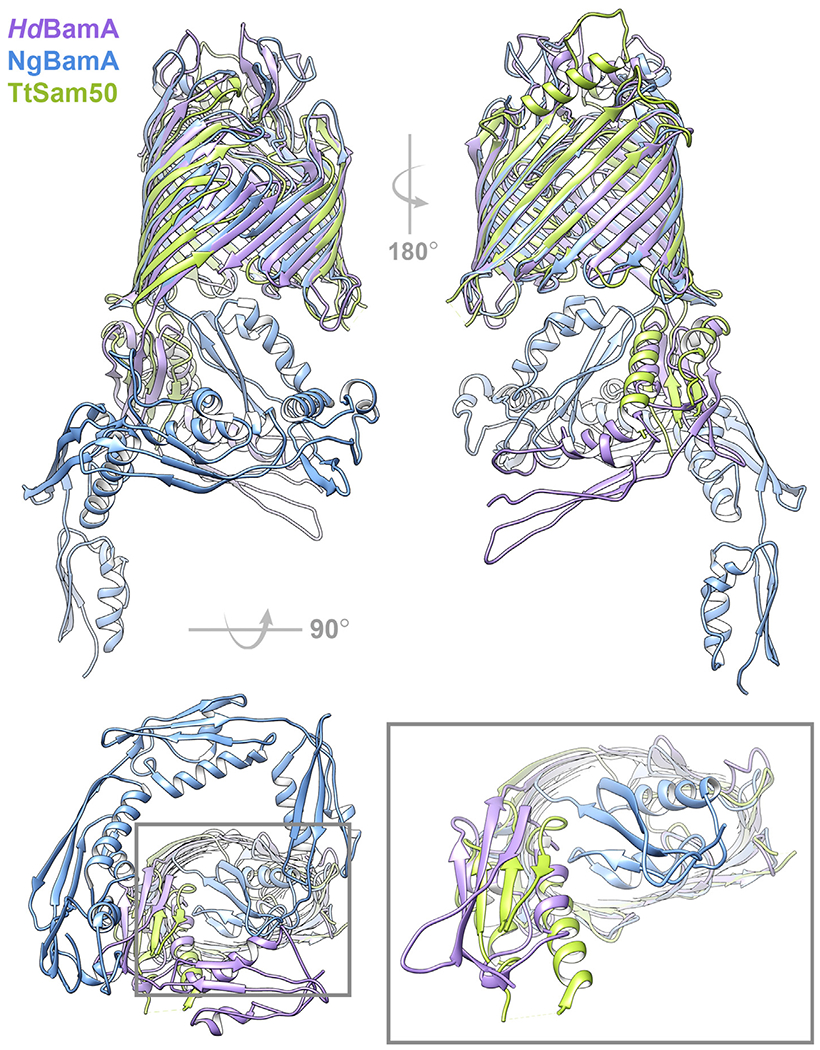
Superposition of BamA and Sam50. Cartoon representation of *Hd*BamA (purple; PDB: 4K3C), *Ng*BamA (blue; 4K3B), and *Tt*Sam50 (green; 6WUT) viewed from the membrane plane showing lateral gate (top left) and back of β-barrel (top right). View from periplasm/IMS (bottom left), gray box inset shows *Ng*BamA POTRA5 occluding the β-barrel lumen while *Hd*BamA POTRA5 and *Tt*Sam50 POTRA domains are oriented away from the β-barrel (bottom right). Superposition generated with ChimeraX v1.1 matchmaker.^183,184^

Sam50 and BamA both contain N-terminal POTRA domain(s) but differ in the number, requirement for activity and proposed functions. The single Sam50 POTRA domain is not essential,^24,100,103^ while BamA contains five or more POTRA domains,^26^ one or two of which are essential depending on the species.^27,29^ The Sam50 POTRA domain is proposed to aid in precursor release from the SAM complex^100,104^ while BamA POTRA domains scaffold the accessory proteins and are implicated in precursor protein binding.^27,131,132^ The BamA POTRA domains are flexible^133,134^ and structures to date contain POTRA domains that either occlude access to the β-barrel lumen or point away from the barrel to allow for access from the periplasm.^40^ *Tt*Sam50 POTRA domain (6WUT) points away from the barrel lumen, similar to *Hd*BamA POTRA 5 (4K3C, Figure 6). In contrast, POTRA 5 of *Ng*BamA is tucked under the β-barrel occluding access (4K3B, Figure 6 inset).

**Figure 7.**
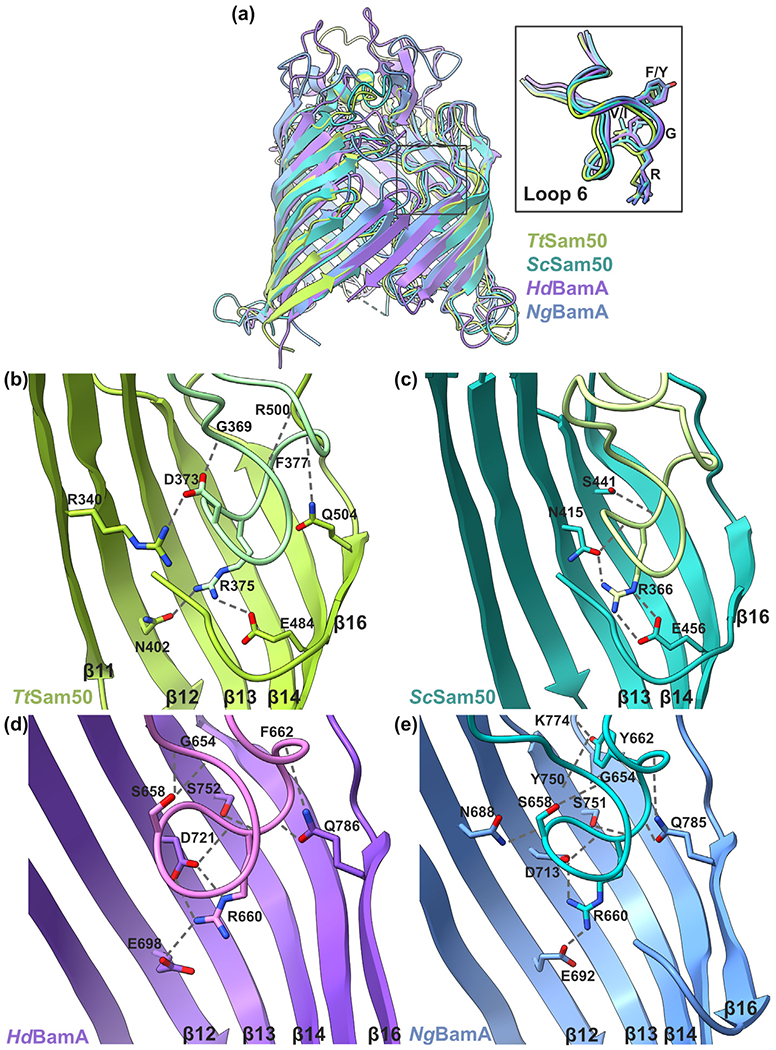
Loop 6 interaction comparison of Sam50 and BamA. (a) β-barrel domain superposition of *Hd*BamA (purple; PDB: 4K3C), *Ng*BamA (blue; 4K3B), *Tt*Sam50 (green; 6WUT), and *Sc*Sam50 (sea green; 7BTW) viewed from the membrane plane in cartoon representation. Grey box indicates cytosolic loop 6, enlarged in inset which contains atom representation for (V/I)RG(F/Y) motif. Loop 6 (V/I)RG(F/Y) motif interactions with β-barrel for (b) *Tt*Sam50 (6WUT) (c) *Sc*Sam50 (7BTW) (d) *Hd*BamA (4K3C), and (e) *Ng*BamA (4K3B). Hydrogen bonds shown as dashed gray lines. Superposition of β-barrel domain generated by ChimeraX v1.1 matchmaker.^183,184^ Additional interactions shown in Supplementary Table 2.

The β-barrel domains of Sam50 and BamA superimpose well, with largest differences noted in the loop conformations and POTRA orientations (6WUT, 7BTW, 4K3B, 4K3C; Table 1, Figures 6 and 7(a)). The cytosolic/extracellular loops fold over the top of each barrel, and loop 6 shares similar conformations between Sam50 and BamA (Figure 7(a)).^30,96,135^ Deletion or mutation of the highly conserved loop 6 (V/I)RG(F/Y) motif results in growth and β-barrel biogenesis defects in both systems.^24,136^ The conserved loop 6 motif interacts with residues in the β-barrel lumen to stabilize the barrel, specifically with β12 and β16 in the *Tt*Sam50, *Ng*BamA, and *Hd*BamA structures. Loop 6 interaction with β11 is unique to *Tt*Sam50 while both *Tt*Sam50 and *Sc*Sam50 contain loop 6 interactions with β15 that are not observed in the *Ng*BamA and *Hd*BamA structures (Figure 7(b)–(e), Supplementary Table 2). *Sc*Sam50, *Ng*BamA and *Hd*BamA structures contain loop 6 interactions with β13 and β14 (Figure 7(c)–(e), Supplementary Table 2). In both *Tt*Sam50 and *Sc*Sam50, loop 6 interacts with β16 residues following the kinking glycine (Supplementary Table 2) which likely helps stabilize the β-strand curled into the barrel lumen.

The β-barrel domains of Sam50 and BamA contain a lateral gate which is closed by varying numbers of hydrogen bonds, from zero in the *Tt*Sam50 and *Sc*Sam50 structures (Figure 4 (b)),^96,99^ to two or eight in *Ng*BamA and *Hd*BamA structures (Supplementary Figure 1).^30^ Differences in number of hydrogen bonds closing the lateral gate are consistent with the requirement of flexibility within the lateral gate for BamA function.^137,138^ Sam50 and BamA both contain a highly conserved glycine within p16 that kinks the strand and aids in lateral gate opening.^112^

Aromatic residue positions of the Sam50 and BamA β-barrel domains reveal that the lateral gate membrane thickness is approximately 11 Å while the membrane thickness on the backside of the barrel is 19.9–22.5 Å. Membrane thinning is not observed at the seam formed by the first and last β-strands of other outer membrane β-barrel proteins, such as OmpF or Tom40 (Supplementary Figure 5, Supplementary Table 3). Membrane thinning at the BamA lateral gate has been proposed to aid β-barrel insertion into the membrane.^30,139,140^ Together, these data suggest that the membrane thinning and local defect of the BamA/Sam50 lateral gate could facilitate β-barrel precursor folding by reducing the energetic barrier for membrane insertion.^30,112,137,141,142^

## Precursor protein targeting

The precise precursor targeting in plant cells with both mitochondria and chloroplasts involves signals that evolved for the specific function. These signals are often localized N-terminally and are cleaved after translocation,^143,144^ but mitochondrial proteins can lack this cleavable signal^145^ (Supplementary Resource 5). In contrast, for β-barrel precursor proteins the signal is the most C-terminal hairpin^100,146,147^ and it also initiates barrel biogenesis by binding to strand β1 of the core barrel in the assembly machinery.^148^ This sequence motif is conserved well enough that BAM and SAM complexes can recognize each other’s precursor proteins and facilitate their folding and insertion into the membrane.^149,150^ Hydrophobicity of the last hairpin is important for mitochondrial targeting,^151^ specifically a hydrophilic residue on the C terminus of the penultimate β-strand.^152^ There is a common motif of N-terminal and C-terminal targeting signals. They rely on the hydrophilic character of amino acid residues precisely localized relative to secondary structural elements, and neither the charge nor the secondary structure is sufficient to signal.

## Mechanism of β-barrel-insertion

BAM complex β-barrel insertion mechanisms can be broken down into two major categories: BAM-assisted and BamA-budding.^40^ In the BAM-assisted mechanism the BAM complex aids the insertion of a partially or fully folded β-barrel by locally destabilizing the membrane. This model is supported by several *in vitro* studies showing increased OMP refolding efficiencies when the membrane is thinned or perturbed.^141,148,153–155^ In a variation of this model, the partially-folded precursor proteins are stabilized by chaperones or other Bam proteins before they are inserted into the membrane.^156,157^

In the BamA-budding mechanism, the precursor proteins with a β-signal and stabilized by chaperones are delivered to BamA. The BamA β-barrel strand 1 serves as a template to the precursor protein that binds to it and starts forming a new OMP barrel by β-augmentation.^19^ Each folded β-strand nucleates a new strand until the new barrel is complete and the β-signal dissociates from the first β-strand of BamA and associates with its own first β-strand in a strand exchange process.

To prevent a super-pore formation in the membrane, the new OMP separates from BamA by ‘budding’ through the open lateral gate of BamA.^46,47^ This model is further supported by a new study in which partially folded BamA precursor proteins were trapped on BamA in the process of β-augmentation by crosslinking.^20^ The β-signal of the folding barrel forms a strong interaction with strand β1 of the BamA β-barrel, whereas the other two edges curl inward and do not pair.^24,158,159^ After the substrate barrel has folded, the rapid sequential replacement of the substrate-BamA H-bonds by substrate–substrate H-bonds seems more favorable than a slow, simultaneous H-bond exchange between these β-strands.^20^

**Figure 8.**
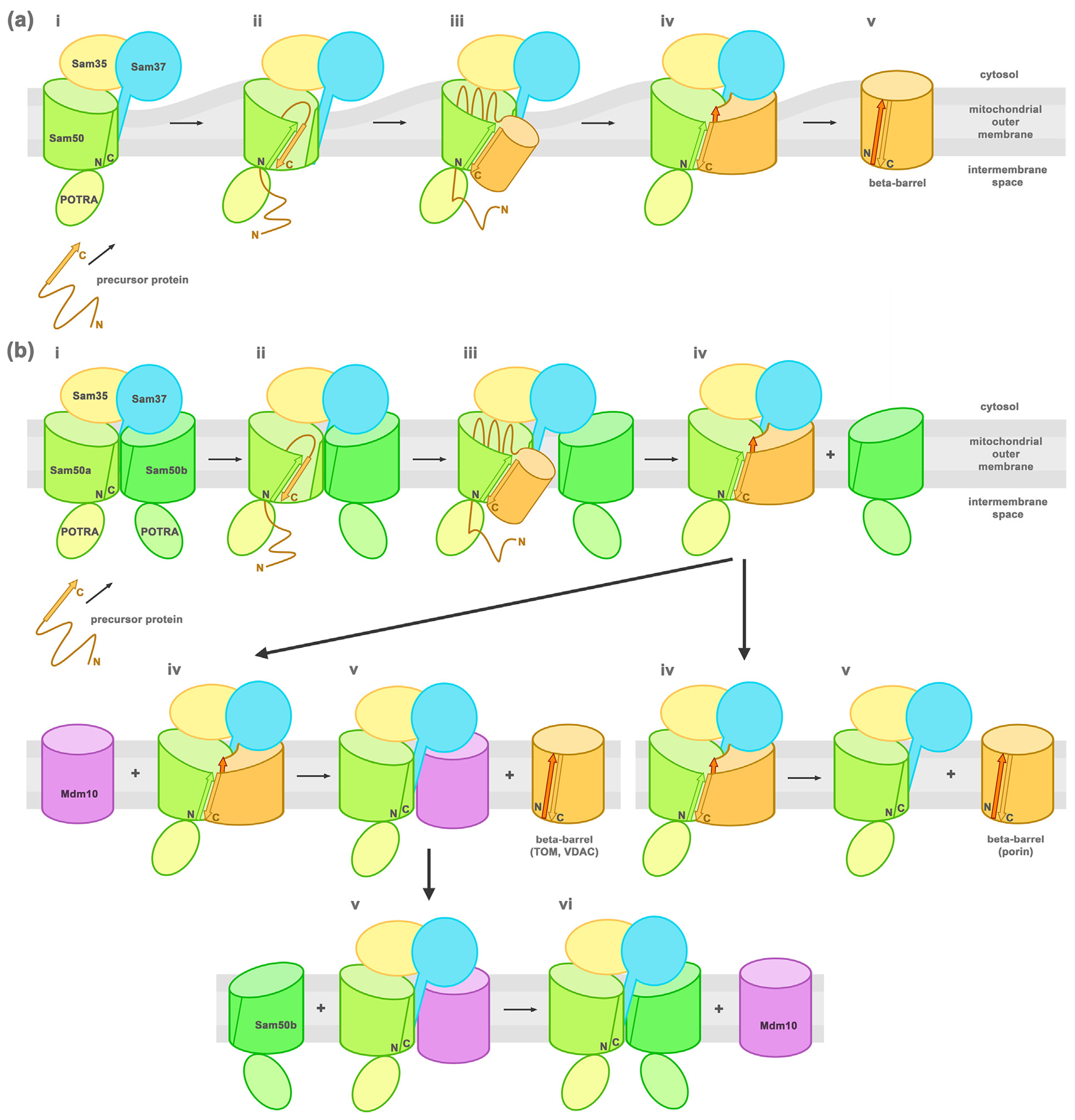
Model of β-barrel assembly mechanism (based on 20,24,99,158). (a) SAM monomer complex lateral gate insertion model (i) Sam50 (green) lateral gate formed by β1-β16 is in a closed state when the precursor protein is recruited to the SAM complex. (ii) The precursor protein β-signal (orange arrow) binds to Sam50 β1 strand (green arrow) displacing β16 and opening the lateral gate. (iii) The precursor protein is sequentially folded through a series of β-hairpin insertion events, building a new β-barrel (orange) that expands the lateral gate further. (iv) Once the last β-strand (dark orange arrow) is incorporated, H-bonds form between the first (orange arrow) and last strands to close the newly folded β-barrel. (v) The new β-barrel is released laterally into the membrane and the Sam50 lateral gate closes. (b) Barrel-swapping model (i) The SAM complex is formed by a SAM monomer + Sam50b second barrel (ii) the precursor protein β-signal binds as in (a). (iii) The folding β-barrel slowly displaces Sam50b. (iv) The fully folded new barrel can follow two paths depending on the protein: (v) Tom40 or VDAC is switched out by Mdm10 (left) and Porin dissociates (right). (vi) The SAM monomer + Mdm10 complex is regenerated by barrel switching with Sam50b.

Based on the SAM complex current data and the BAM complex models, a lateral gate insertion model has been proposed for the SAM complex (Figure 8(a)). In this model, the Sam50 lateral gate opens to allow the precursor protein β-signal to interact with Sam50 β1. The remainder of the precursor protein is sequentially folded through a series of β-hairpin insertion events, which expand the lateral gate further, then the new β-barrel is released laterally into the outer membrane and the Sam50 lateral gate closes.^20,24,158^ Membrane thinning around the lateral gate likely facilitates destabilization of this region and β-signal binding to β1. The β-signal possibly dislocates strand β16 of the lateral gate, a process energetically more favorable than lateral gate opening and then binding the β-signal. Lateral release of the folded barrel into the membrane might also be facilitated by the thinner membrane around the lateral gate.

Based on the *Sc*SAM complex structures a new barrel-switching mechanism was put forward (Figure 8(b)).^99^ The precursor protein would bind Sam50a in the SAM monomer + Sam50b complex and follow the budding mechanism. When the newly folded barrel reaches a certain size, it can dislocate the Sam50b barrel and take its place under Sam37. Then the completed barrel can be released by different mechanisms depending on the folded β-barrel protein. High abundance porin would dissociate spontaneously from the SAM core complex, while lower abundance Tom40 barrels would be dislocated and released by an Mdm10 barrel. Mdm10 from the SAM monomer + Mdm10 complex would dissociate or would be displaced by another Sam50 barrel (Sam50b) to regenerate the SAM monomer + Sam50b complex. Sam50b would act as a placeholder in this complex.

If we consider all available data, the energetics of these processes are not clear. The interactions between Sam37 and Sam50b do not seem to be favorable, since helices 6–8 of Sam37 are unstructured in the SAM monomer + Sam50b structure, due to the cytosolic loops of Sam50b. The same region of Sam37 is partially helical in the SAM monomer + Mdm10 structures, and it dives into the Mdm10 barrel for a more stable interaction. Sam37 helices 6–8 are less hydrophobic than a TM helix and their folding would be more favorable inside a barrel than the membrane (Supplementary Figure 6). This more extensive interaction is also possible with other mitochondrial β-barrels (Mdm10, VDAC/porin, Tom40) that do not have cytosolic loops obstructing Sam37. This observation questions the spontaneous release of newly folded porin barrels from the SAM monomer, since Sam37 helices would dive into the porin barrel, stabilizing its interaction. The same is true for the SAM monomer + Mdm10 complex.

Another result from the *Sc*SAM structures is the different relative abundance of Sam37 in the presence of different length precursor proteins folding on the SAM complex. In the presence of short precursor proteins (7 strands) there is a high abundance of Sam37 in the complex (1.1:1 stoichiometry with Sam35), whereas in the presence of long precursor proteins (barrel missing 1 strand) the abundance is almost half (0.6:1 relative to Sam35).^99^ This would imply that Sam37 is present when the precursor protein binds the complex and folds more than halfway, then it might dissociate either alone or together with the newly folded barrel. Once the newly folded barrel pushes out the placeholder Sam50b, the Sam37 disordered helical region might guide the folding of the new barrel and extend into it. With the new barrel folded, the Sam37 helices reaching into the barrel would have to become disordered for the barrel to be switched out by other barrels in an energetically favorable way. Otherwise, it might dissociate together with the new barrel from the SAM complex.

The bacterial outer membrane contains many different types of β-barrel proteins, which are formed by 8–36 β-strands.^160,161^ In contrast, the fungal mitochondrial outer membrane contains only four different β-barrels which are either 16 β-strands (Sam50) or 19 β-strands (Tom40, VDAc/Porin, Mdm10).^115,162^ The large variation of bacterial β-barrel proteins suggest the possibility of multiple different folding and insertion mechanisms depending on the precursor protein, whereas the similarity of Sam50 precursor proteins suggests one or two mechanisms.

## Perspectives

A common feature across all outer membranes containing β-barrel proteins is the presence of a specialized machinery for β-barrel biogenesis. There are some common characteristics of the β-barrel biogenesis shared across species: (a) insertion occurs from the inner side of the membrane, (b) soluble chaperones in the periplasm or IMS aid the process and (c) the core protein in the assembly machinery complex is well conserved. BamA has homologues across all Gram-negative bacteria and in the outer membrane of mitochondria and chloroplasts.^163^ These structures all have POTRA domains followed by a C-terminal β-barrel. While the barrel is highly conserved, the number of POTRA domains varies from one in Sam50 to up to seven in BamA from *Myxococcus*. A single POTRA domain might be the minimum requirement for function and additional domains might have been gained during evolution. The number of POTRA domains seems to correlate with the number and size of β-barrel proteins in the cell,^29,143,164^ with mitochondria and chloroplasts harboring very few β-barrel proteins in contrast to bacteria. The orientation of POTRA domains is another characteristic common for all types of β-barrel assembly machineries with recent studies confirming that the POTRA domain of Oep80 also points toward the IMS.^130^

Another proof of the highly homologous nature of these machineries is the ability of one system to fold and assemble barrel proteins from another system. For example, bacterial YadA can be assembled and inserted into the membrane in mitochondria,^165^ or mitochondrial VDAC1 in the *E coli* bacterial outer membrane^150^ and chloroplast Oep37, Oep24 into mitochondria from yeast cells^166^ by the machineries of the respective outer membranes.

A distinguishing feature of the β-barrel assembly machineries is the nature of their accessory proteins. While the BAM complex employs a series of lipoproteins bound to BamA on the periplasmic side of the membrane, the SAM complex has cytoplasmic accessory proteins located on the opposite side of the membrane. The chloroplast Oep80 accessory proteins have not been identified. A more detailed study of the role of accessory proteins is still needed to better understand the β-barrel assembly machineries.

The folding state of the precursor proteins as they are transferred to the β-barrel assembly machinery is currently unknown. It is unclear if this state is the same in bacteria, mitochondria and chloroplasts. If we assume that the five or more POTRA domains in bacterial BAM complexes have a chaperone role, the mitochondrial and chloroplast Sam50 and Oep80 with a single POTRA domain would imply a precursor to exist in a more advanced folding state in eukaryotic systems. Alternatively, the small TIM chaperones might form a supercomplex with the SAM complex while the precursor is passed on, omitting the need for the assistance of a larger number of POTRA domains to prevent the aggregation of the precursor protein.

The POTRA domains of bacteria and mitochondria are proposed to have different functions, with the bacterial POTRA domains involved in precursor protein recognition^27,131,132^ and the mitochondrial POTRA domain involved in precursor release.^100,104^ The contrasting functions of these domains leads us to question how the bacterial precursor proteins are released from the BAM complex. Do the bacterial POTRA domains also aid in precursor release or is a different domain or subunit responsible for release? In mitochondria, Sam37 is also proposed to aid in precursor release^93,100^ and with the interaction of Sam37 and Sam50 POTRA domain in *Tt*SAM^96^ it is tempting to speculate that these two subunits may work together to facilitate precursor release. However, Sam37 is not predicted to contain transmembrane domains in all species^91,96,105–107^ (Supplementary Figure 3, Supplementary Table 1) and therefore must somehow be able to promote precursor release from the cytosol. Sam37 helices 6–8 can more extensively interact with newly formed barrels in the place of Mdm10. However, this interaction seems to stabilize the precursor in its current position and not its release.

While the BAM complex is relatively well characterized in *E. coli*, structures of the BAM complex from other organisms would contribute to the better understating of the intricacies of Bam protein interactions within the complex and the overall mechanism. Despite the variable number of BamA POTRA domains, the bacterial machinery seems to be the most conserved, with no known examples of multiple homologues fulfilling the same function (there is only one BamA, BamB, etc. in the bacterial cell).

The structure and function of the human SAM complex requires further investigation, as the majority of studies to date focus on the SAM complex from yeast and other lower eukaryotes. While it is generally accepted that in higher eukaryotes Metaxin 1 and Metaxin 2 fulfill the functional roles of Sam37 and Sam35, respectively, the sequence conservation between the Metaxins and Sam37 and Sam35 is low (Supplementary Figures 3 and 4).^167^ While the Metaxins are predicted to contain a GST-like fold,^106,107^ like Sam35 and Sam37, it is still unclear how the Metaxins associate with each other and human Sam50. Sam35 and Sam37 interactions are not highly conserved even between the available fungal structures (Supplementary Figures 3 and 4). Considering that the Metaxins are unable to complement *S. cerevisiae* Sam37 and Sam35 deletion mutants,^106,167^ it is likely that Metaxin 1 and Metaxin 2 associate with each other and with human Sam50 through different interactions. The human SAM complex structure should address these gaps in knowledge, but biochemical characterization of the subunit interactions through truncation pull-down experiments would also provide valuable information.

Additionally, very little is known about the role of SAM complex mutations in human disease. Single-nucleotide polymorphisms in the *Sam50* gene have been associated with susceptibility to nonalcoholic fatty liver disease in Chinese, Korean, and Japanese populations,^168–170^ however more work is required at the protein level to identify how these variants influence the SAM complex structure and function. Mandibuloacral dysplasia progeroid syndrome patients have Metaxin 2 gene mutations that result in loss of Metaxin 2 protein expression, Metaxin 1 depletion, mitochondrial network fragmentation, and impaired apoptosis.^171^ A human SAM complex structure will advance understanding of how Metaxin 2 facilitates Metaxin 1 localization to the mitochondrial outer membrane.

From the three analogous barrel-assembly machineries in bacteria, mitochondria and chloroplasts, the least well characterized is the chloroplast system. The mitochondrial and chloroplast machineries have different evolutionary origins, but they converged toward the same function. The exact components of the complex are not all known and structurally characterized. This can be attributed to the higher complexity of these systems in chloroplasts, with multiple homologs for the same protein and more variation among paralogs from different species.

Several inhibitors of BamA have been identified.^172–175^ One of these inhibitors, darobactin, is thought to inhibit BAM complex function by binding to the lateral gate of BamA, since mutations in this region result in darobactin resistance.^173^ Two different antimicrobial peptides have been shown to bind BamA extracellular loops and have bactericidal activity.^174^ Another compound, IMB-H4, inhibits BAM complex function by binding to BamA and preventing BamD association.^175^ It will be important to study the effects of these inhibitors on the function of the SAM complex, to ensure the therapeutics designed to treat bacterial pathogens do not have adverse effects on Eukaryotic cells. These studies may also help to identify differences between functional mechanisms in the BAM and SAM complexes.

The major bottleneck in advancing the understanding of β-barrel biogenesis is the availability of the proteins involved in these processes. These membrane proteins are difficult to overexpress and purify in adequate quantities for structural studies. The recent widespread adoption of atomic-resolution cryo-EM permitted the solution of several large and dynamic multiprotein complex structures involved in the biogenesis process, complexes that perhaps were not yielding diffraction-quality crystals before. While X-ray crystallography has already reached relative maturity, cryo-EM methodologies and instrumentation still develop at a rapid pace. This is especially true for sample preparation techniques, like grid preparation from very small amounts of protein, that can enable the structural study of much more elusive complexes. Recent advances in cryo-EM data collection hardware have led to ultra-high resolution structures of GABA_A_-β3 receptor and apoferritin (1.7 Å and 1.2 Å, respectively).^176–178^ With these advances in energy filters, detectors and other hardware, it is very likely that higher resolution structures of the β-barrel biogenesis machinery are attainable. Cryo-EM tomography opens the possibility of studying these complexes in their native environment, in the membrane of isolated mitochondria for example, with all of the other components present. Time-resolved microscopy could characterize different steps in the biogenesis process, with freeze-trapped intermediates. In the not-too-distant future, we expect to have a much better understanding of precisely how β-barrel proteins fold.

## Supplementary Material

1

2

## Figures and Tables

**Table 1:** Available structures discussed in the text.

Molecule	Organism	Res	Method	PDB
**Bam proteins and BAM complexes**				
Bam ACDE^17^	*Escherichia coli*	3.39	X-ray	5EKQ
Bam ABCDE^15^	*Escherichia coli*	2.9	X-ray	5D0O
Bam ACDE^15^	*Escherichia coli*	3.5	X-ray	5D0Q
Bam ABCDE^16^	*Escherichia coli*	3.56	X-ray	5AYW
Bam ABCDE^18^	*Escherichia coli*	4.9	Cryo-EM	5LJO
Bam ABCDE^19^	*Escherichia coli*	3.05	X-ray	6LYS
BamA-β12^OmpLA^BCDE^19^	*Escherichia coli*	3.19	X-ray	6LYQ
BamA- β8^OmpA^BCDE^19^	*Escherichia coli*	3.28	X-ray	6LYR
Bam ABCDE in nanodisc^19^	*Escherichia coli*	4.2	Cryo-EM	6LYU
Bam ABCDE + substrate^20^	*Escherichia coli K12*	4.1	Cryo-EM	6V05
BAM ABCDE in nanodisc [Iadanza unpublished]	*Escherichia coli*	9.8	Cryo-EM	6SN9
Bam A (barrel only)^135^	*Escherichia coli*	2.6	X-ray	4N75
Bam A (barrel only)^185^	*Escherichia coli O157:H7*	2.6	X-ray	6FSU
Bam A (barrel only)^186^	*Salmonella enterica*	2.92	X-ray	5OR1
Bam A (POTRA5)^140^	*Escherichia coli*	3.0	X-ray	4C4V
Bam A (POTRA45)^30^	*Haemophilus ducreyi*	2.91	X-ray	4K3C
POTRA45 (BamA)^134^	*Escherichia coli*	2.69	X-ray	3OG5
POTRA45 (BamA)^187^	*Escherichia coli*	1.5	X-ray	3Q6B
POTRA12 (BamA)^131^	*Escherichia coli*	–	NMR	2V9H
POTRA1234 (BamA)^27^	*Escherichia coli*	2.7	X-ray	2QCZ
POTRA1234 (BamA)^27^	*Escherichia coli*	2.2	X-ray	2QDF
POTRA1234 (BamA)^132^	*Escherichia coli*	3.3	X-ray	3EFC
Bam A (full length)^30^	*Neisseria gonorrhoeae*	3.2	X-ray	4K3B
Bam B^33^	*Escherichia coli*	1.65	X-ray	3Q7M
Bam B^33^	*Escherichia coli*	1.77	X-ray	3Q7N
Bam B^33^	*Escherichia coli*	2.09	X-ray	3Q7O
Bam B^188^	*Escherichia coli*	2.6	X-ray	2YH3
Bam B^189^	*Escherichia coli*	2.0	X-ray	3Q54
Bam B^190^	*Escherichia coli*	1.8	X-ray	3PRW
Bam B^191^	*Escherichia coli*	2.6	X-ray	3P1L
Bam B^34^	*Pseudomonas aeruginosa*	1.85	X-ray	4HDJ
Bam B [Agnew unpublished]	*Moraxella catarrhalis*	2.33	X-ray	4IMM
Bam C (N terminus)^188^	*Escherichia coli*	1.55	X-ray	2YH6
Bam C (N terminus)^192^	*Escherichia coli*	–	NMR	2LAF
Bam C (C terminus)^36^	*Escherichia coli*	1.5	X-ray	3SNS
Bam C (C terminus)^192^	*Escherichia coli*	–	NMR	2LAE
Bam D^188^	*Escherichia coli*	1.8	X-ray	2YHC
Bam D^41^	*Escherichia coli K12*	2.6	X-ray	3Q5M
Bam D^42^	*Rhodothermus marinus*	2.15	X-ray	3QKY
Bam D^193^	*Neisseria gonorrhoeae*	2.5	X-ray	5WAQ
Bam E^188^	*Escherichia coli*	1.8	X-ray	2YH9
Bam E^193^	*Neisseria gonorrhoeae*	2.45	X-ray	5WAM
Bam E^194^	*Escherichia coli*	–	NMR	2KM7
Bam E^195^	*Escherichia coli*	–	NMR	2KXX
Bam A (POTRA35) B^35^	*Escherichia coli*	2.15	X-ray	4XGA
Bam A (POTRA35) -B (fusion)^196^	*Escherichia coli*	3.1	X-ray	4PK1
Bam A (POTRA45) -B (fusion)^197^	*Rhodothermus marinus*	2.0	X-ray	5EFR
Bam CD^37^	*Escherichia coli*	2.9	X-ray	3TGO
**Bacterial chaperones**				
SurA^198^	*Escherichia coli*	3.0	X-ray	1M5Y
Skp^199^	*Escherichia coli*	2.3	X-ray	1U2M
Skp^200^	*Escherichia coli*	2.35	X-ray	1SG2
DegP*^201^	*Escherichia coli*	2.8	X-ray	1KY9
**SAM complexes**				
SAM monomer in nanodisc^96^	*Thermothelomyces thermophilus*	3.4	Cryo-EM	6WUH
SAM dimer 1^96^	*Thermothelomyces thermophilus*	3.2	Cryo-EM	6WUL
SAM monomer from 1^96^	*Thermothelomyces thermophilus*	3.0	Cryo-EM	6WUT
SAM dimer 2^96^	*Thermothelomyces thermophilus*	3.6	Cryo-EM	6WUM
SAM dimer 3^96^	*Thermothelomyces thermophilus*	3.9	Cryo-EM	6WUN
SAM monomer^96^	*Thermothelomyces thermophilus*	3.7	Cryo-EM	6WUJ
SAM monomer + Sam50b^99^	*Saccharomyces cerevisiae*	2.9	Cryo-EM	7BTW
SAM monomer + Mdm10^99^	*Saccharomyces cerevisiae*	2.8	Cryo-EM	7BTX
SAM monomer + Mdm10 in nanodisc^99^	*Saccharomyces cerevisiae*	3.2	Cryo-EM	7BTY
**Tom proteins and TOM complexes**				
Tom5,6,7,22,40 dimer^66^	*Saccharomyces cerevisiae S288C*	3.06	Cryo-EM	6UCU
Tom5,6,7,22,40 tetramer^66^	*Saccharomyces cerevisiae S288C*	4.1	Cryo-EM	6UCV
Tom5,6,7,22,40 dimer^67^	*Saccharomyces cerevisiae*	3.81	Cryo-EM	6JNF
Tom5,6,7,22,40 dimer^68^	*Homo sapiens*	3.4	Cryo-EM	7CK6
Tom5,6,7,22,40 dimer^65^	*Neurospora crassa*	6.8	Cryo-EM	5O8O
Tom70 (cytosolic)^64^	*Saccharomyces cerevisiae*	3.0	X-ray	2GW1
Tom20 (cytosolic)^63^	*Rattus norvegicus*	–	NMR	1OM2
**TIM complexes**				
TIM 8/13^202^	*Saccharomyces cerevisiae*	2.6	X-ray	3CJH
TIM 9/10^203^	*Homo sapiens*	3.3	X-ray	2BSK
TIM 9/10^204^	*Saccharomyces cerevisiae*	2.5	X-ray	3DXR

* Only the highest resolution structure of DegP from the PDB was included in this table.

## Abbreviations:

OMP: outer membrane protein
OM: outer membrane
MOM: mitochondrial outer membrane
OEM: chloroplast outer envelope membrane
IMS: intermembrane space
POTRA domain: polypeptide transport-associated domain
TM: transmembrane
TPR: tetratricopeptide repeat
ERMES: endoplasmic reticulum-mitochondria encounter structure
cTP: chloroplast transit peptide
BAM: barrel-assembly machinery
TOM: translocase of the outer membrane
VDAC: voltage-dependent anion-selective channel
TIM: translocase of the inner membrane
SAM: sorting and assembly machinery
MDM: mitochondrial distribution and morphology
MIM: mitochondrial import complex
TOC: translocase of the outer membrane of chloroplast
Oep80: outer envelope protein 80
cryo-EM: cryo-electron microscopy
