# Supplementary material for: Building Better Barrels – β-barrel Biogenesis and Insertion in Bacteria and Mitochondria
Source: J Mol Biol. Author manuscript; Available in PMC 2021 Aug 6. (PMC8292188; doi:10.1016/j.jmb.2021.166894)
Supplement: 1 [file NIHMS1684439-supplement-1.pdf]

Supplementary Information

Building better barrels - β-barrel biogenesis and insertion in bacteria and mitochondria

Kathryn A. Diederichs, Susan K. Buchanan, and Istvan Botos*

Laboratory of Molecular Biology, National Institute of Diabetes & Digestive & Kidney Diseases, National Institutes of Health, 9000 Rockville Pike, Bethesda, MD 20892 USA.

*Correspondence: Istvan Botos (botosi@niddk.nih.gov)

Supplementary Resource 1

SurA

The core of the SurA structure is formed by the N-terminal, C-terminal and first peptidyl-prolyl isomerase (PPIase) domains. The second PPIase is tethered ~30Å from the core, forming a dumbbell-shaped overall structure (Figure 1a). Peptides can bind into the extended crevice of the core module [1]. SurA chaperones the vast majority of outer membrane proteins [2].

Skp

Skp is a homotrimeric 17kDa protein with a jellyfish-like shape, where α-helical tentacles protruding from a core formed by intersubunit β-sheets enclose a central cavity (Figure 1a). The central cavity can bind precursor proteins while the exterior of the molecule can interact with membrane lipids and LPS. Skp has a similar overall shape to the cytosolic chaperone prefoldin from eukaryotes and archaea [3,4]. Skp, together with DegP, rescues outer membrane proteins that fall off the SurA pathway [5].

DegP *(also known as HtrA)*

The 49kDa DegP was first identified as a serine protease [6] and later shown to also have chaperone activity [7]. The DegP structure (1KY9, Table 1) is a monomer with three domains: a protease domain followed by two PDZ domains. It forms hexamers with staggered association of trimeric rings, in which proteolytic sites face a central cavity that is accessible only laterally (Figure 1a). The cavity is lined by hydrophobic patches where unfolded polypeptides can bind. When DegP acts as a chaperone, its protease domain is in an inactive state [8].

Upon precursor protein binding, both SurA and Skp undergo conformational changes and dynamic re-organization. Moreover, precursor dynamics on the chaperone surface were also shown to be crucial for function [9]. Both chaperones are likely to interact with the outer membrane and BamA. While SurA is the primary chaperone in *E. coli*, in its absence most precursors can use Skp efficiently [10].

Supplementary Resource 2

TIM8/13 and TIM9/10

The high-resolution structures of these soluble chaperones reveal alternating circular hexamers of TIM8/13 and TIM9/10, where each six-bladed α-propeller contains three molecules of the respective TIM protein (Figure 1b) [11–13]. Multiple clamp-like binding sites with conserved hydrophobic residues hold the precursor proteins in an elongated form that are translocation-competent. Upon precursor protein binding, the structures of the chaperones remain intact. The chaperone to precursor stoichiometry depends on the size of the precursor, more hexameric complexes being used for larger proteins [14]. TIM8/13 uses salt bridges to interact with the hydrophilic part of a substrate protein and has weaker interactions with the transmembrane part than TIM9/10, and therefore has a broader specificity than TIM9/10 for amphipathic proteins. TIM9/10 outcompetes TIM8/13 in binding hydrophobic precursors [15].

The soluble TIM8/13 complex in the IMS has overlapping functions with the TIM9/10 complex [16,17]. Both α-helical and β-barrel polypeptide precursors bind to the same binding clefts of the chaperones. While the β-barrel precursors for VDAC and Tom40 depend on both chaperones, the transport of metabolite carrier Aac2 and carrier-like precursor TIM23 depend mainly on TIM9/10 [12,18,19]. Barrel precursor proteins need to have a β-hairpin conformation to be able to bind TIM chaperones, a conformation ideal for β-barrel protein insertion by the SAM complex [20]. This might suggest that the POTRA domain is not as important in mitochondrial β-barrel biogenesis as for the equivalent bacterial process, where β-barrel precursors are unstructured [21–23]. It has yet to be determined if the TIM chaperones form supercomplexes with the TOM complex and/or SAM complex to prevent precursor aggregation following import and to maintain the precursor β-hairpin conformation for SAM complex binding.

Supplementary Resource 3

The Toc75 proteins of chloroplasts and cyanobacteria belong to a different branch of the Omp85 and Sam50 (Tob55) family [24,25]. In *Arabidopsis thaliana,* five different isoforms (I-V) of Toc75 were identified [26].

Toc75 *(or AtToc75-III in Arabidopsis)*

Toc75 is the pore-forming subunit of the TOC complex and is functionally equivalent to mitochondrial Tom40 [27]. It is highly abundant in the outer membrane and is synthesized in the cytoplasm. Toc75 is predicted to contain three IMS-facing N-terminal POTRA domains and a transmembrane C-terminal 16-strand β-barrel. The X-ray structure of the three POTRA domains shows a conserved architecture, with POTRA2 containing an additional elongated α-helix not observed in other POTRA domains [28]. It is suggested that these POTRA domains have a chaperone-like function, preventing the misfolding or aggregation of the translocated preprotein.

Toc159 and Toc34

These receptor proteins of the TOC complex contain GTPase domains that control preprotein binding and translocation. Both proteins are C-terminally anchored in the chloroplast outer membrane (OEM) directing their GTP-binding domains toward the cytosol [29]. Together with Toc75 they form the heterotrimeric TOC core complex. Toc159 contains three functional domains: an intrinsically disordered acidic domain, the GTPase domain and the 54 kDa membrane-anchor domain [30,31]. *Arabidopsis* has three additional AtToc159 homologues: AtToc134, AtToc120 and AtToc90 which mainly differ in their disordered domains. AtToc159 is the most abundant isoform [32]. Toc34 has a cytosolic GTPase domain and is anchored in the membrane by a single transmembrane helix. *Arabidopsis* expresses the additional AtToc33 in young tissues besides AtToc34 expressed at low levels in all organs during development [33]. Toc159 and Toc34 bind different regions of a preprotein cTP signal and they could act simultaneously [34].

Toc64

Toc64 with three tetratricopeptide repeats has a cytosolic receptor site for Hsp90/70 and likely functions as a receptor for plastid protein import [35]. It is homologous to Tom70 from mitochondria but is not associated

with the TOC complex. Toc64 can deliver preproteins to Toc34 but is not essential for transport, instead it could act as an additional regulatory component to the TOC complex [36].

Oep80 *(or AtOep80, AtToc75-V in Arabidopsis)*

Oep80 is not a member of the TOC complex, but essential for viability in *Arabidopsis* [37]. It was suggested to be involved in β-barrel insertion into the membrane, but to date there is no direct evidence supporting this claim. *At*Toc75-III and *At*Toc75-V (Oep80) belong to two different branches of the Omp85 superfamily [38]. Gene duplication in an early eukaryotic ancestor produced two clades: while Oep80 (*At*Toc-V) retained the original function of the cyanobacterial protein, *At*Toc75-III rapidly evolved into a protein import channel [39].

The specific signals for targeting β-barrel proteins to the chloroplast are still unknown, but the precursor of Toc75-III follows a unique pathway. It is synthesized with an N-terminal extension which functions as a dual targeting signal and is cleaved during maturation [40]. The first portion of the signal targets the stroma where is cleaved, while the second portion functions as a stop-transfer signal that prevents full translocation across the inner membrane [41]. The TOC complex seems to be the equivalent machinery of the mitochondrial TOM complex, which translocates precursor proteins into the IMS. Translocated β-barrel precursor proteins are then possibly integrated into the membrane by Oep80. The exact cytosolic components and mechanism for sorting, targeting and insertion of β-barrel proteins into the chloroplast outer envelope membrane are still unknown [42].

Supplementary Resource 4

Multiple populations of the SAM complex in detergent were observed during cryo-EM data processing [43]. First, there was a monomeric population containing one copy of each SAM complex subunit, that was very similar to the lipid nanodisc monomer. The majority of particles were dimers, composed of two copies each of Sam50, Sam37, and Sam35. Three different populations of dimers were observed, all of which were non-physiological dimers with the SAM complexes in an up-down orientation. Biochemical topology experiments with the SAM complex in mitochondria do not support this association [44–47], therefore dimerization possibly occurred upon membrane solubilization with detergent or downstream purification steps. The monomer extracted from dimer 1 was highly similar to the detergent monomer and lipid nanodisc conformations. In contrast, the other two dimer populations contained Sam50 subunits with β1- β4 rotated outwards approximately 45 degrees, ultimately resulting in a β1- β1 interaction between the two Sam50 copies. While the dimer populations are non-physiological likely due to the presence of detergent [48], the flexibility of β1-4 supports the ability to accommodate β-barrel precursor protein at the lateral gate (Supplementary Movie 1).

Supplementary Resource 5

In mitochondria the signal length is on average 50 residues [49], with 80 residues before a folded domain required for efficient translocation [50]. In chloroplasts this is 60 residues and can also include the N-terminus of the first domain [51]. These residues in mitochondria are more basic, with a lower hydrophobicity than found in chloroplasts [52], and can form an amphiphilic α-helix [53]. Chloroplast signals can contain motifs like FPxxRK or FGLK [54,55], while for the 19 N-terminal residues in mitochondria the neighboring residues are mutually dependent [56]. The specific signal for targeting β-barrel proteins in chloroplasts remains to be established.

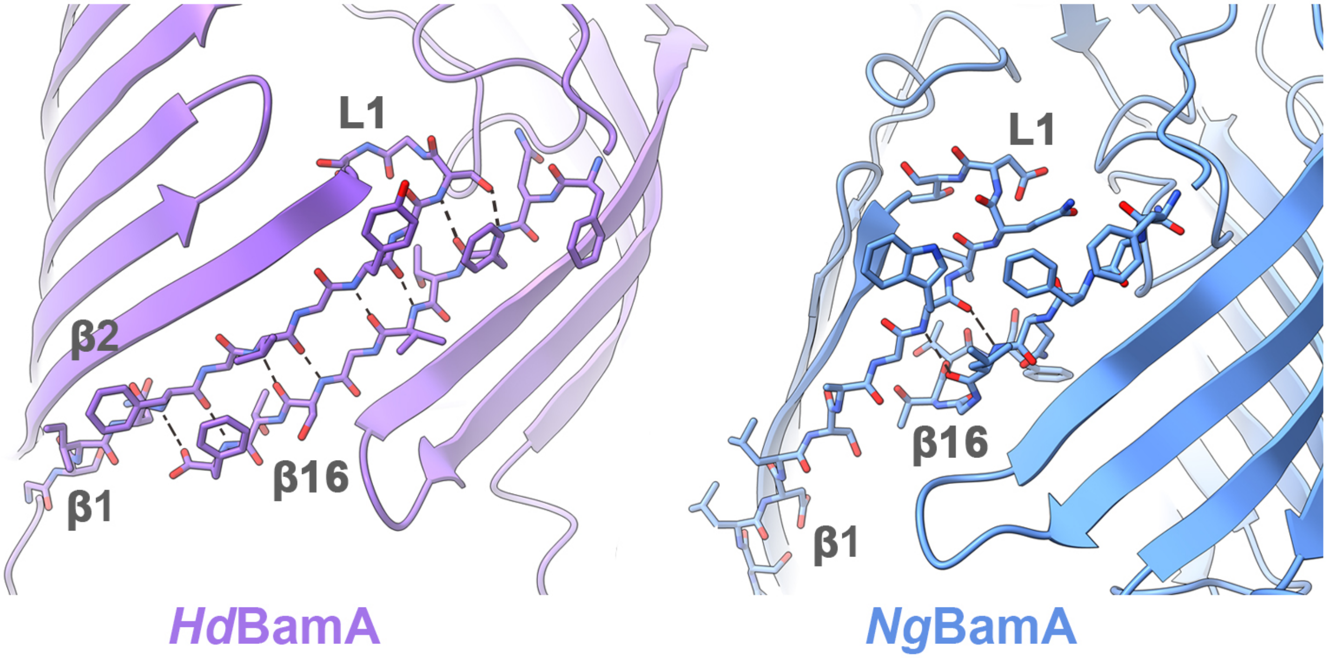

Supplementary Figure 1. BamA lateral gate interactions. The structure of the lateral gate in *Hd*BamA (4K3C) and *Ng*BamA (4K3B). Hydrogen bonds are shown as dashed grey lines.

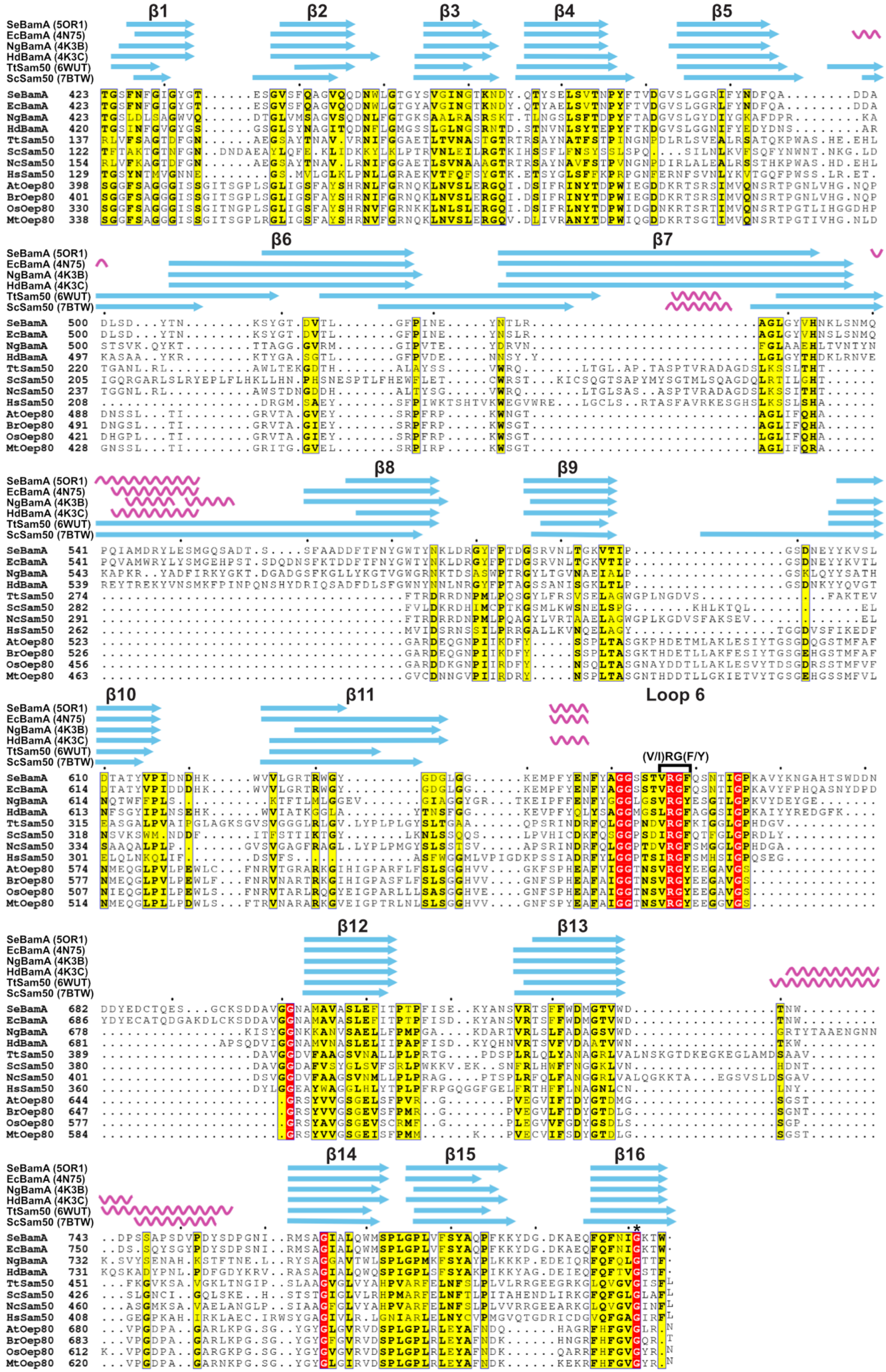
β1
β2
β3
β4
β5
β6
β7
β8
β9
β10
β11
Loop 6
(V/I)RG(F/Y)
β12
β13
β14
β15
β16
SeBamA (5OR1)
EcBamA (4N75)
NgBamA (4K3B)
HdBamA (4K3C)
TtSam50 (6WUT)
ScSam50 (7BTW)
SeBamA
EcBamA
NgBamA
HdBamA
TtSam50
ScSam50
NcSam50
HsSam50
AtOep80
BrOep80
OsOep80
MtOep80

Supplementary Figure 2. Structure-based sequence alignment of BamA/Sam50/Oep80 β-barrel domains. Blue arrows represent β-strands, magenta waves represent α-helices. Asterisk identifies conserved glycine that kinks β16. Alignment generated by T-Coffee Expresso [57–61], adjusted with JalView [62], and colored with ESPript 3.0 [63] based on percent equivalent and 0.6 global score. Se: *Salmonella enterica* subspecies *typhimurium* (UniProt Q8ZRP0; PDB 5OR1), Ec: *Escherichia coli* (UniProt P0A940; PDB 4N75), Ng: *Neisseria gonorrhoeae* (UniProt Q5F5W8; PDB 4K3B), Hd: *Haemophilus ducreyi* (UniProt Q93PM2; PDB 4K3C), Tt: *Thermothelomyces thermophilus* (UniProt G2QFF9; PDB 6WUT), Sc: *Saccharomyces cerevisiae* (UniProt P53969), Nc: *Neurospora crassa* (UniProt V5IKW7), Hs: *Homo sapiens* (UniProt Q9Y512), At: *Arabidopsis thalian*a (UniProt Q9C5J8), Br: *Brassica rapa subsp. pekinensis* (UniProt M4CDG9), Os: *Oryza sativa subsp. Japonica* (UniProt Q6H7M7), Mt: *Medicago truncatula* (UniProt G7IUC7).

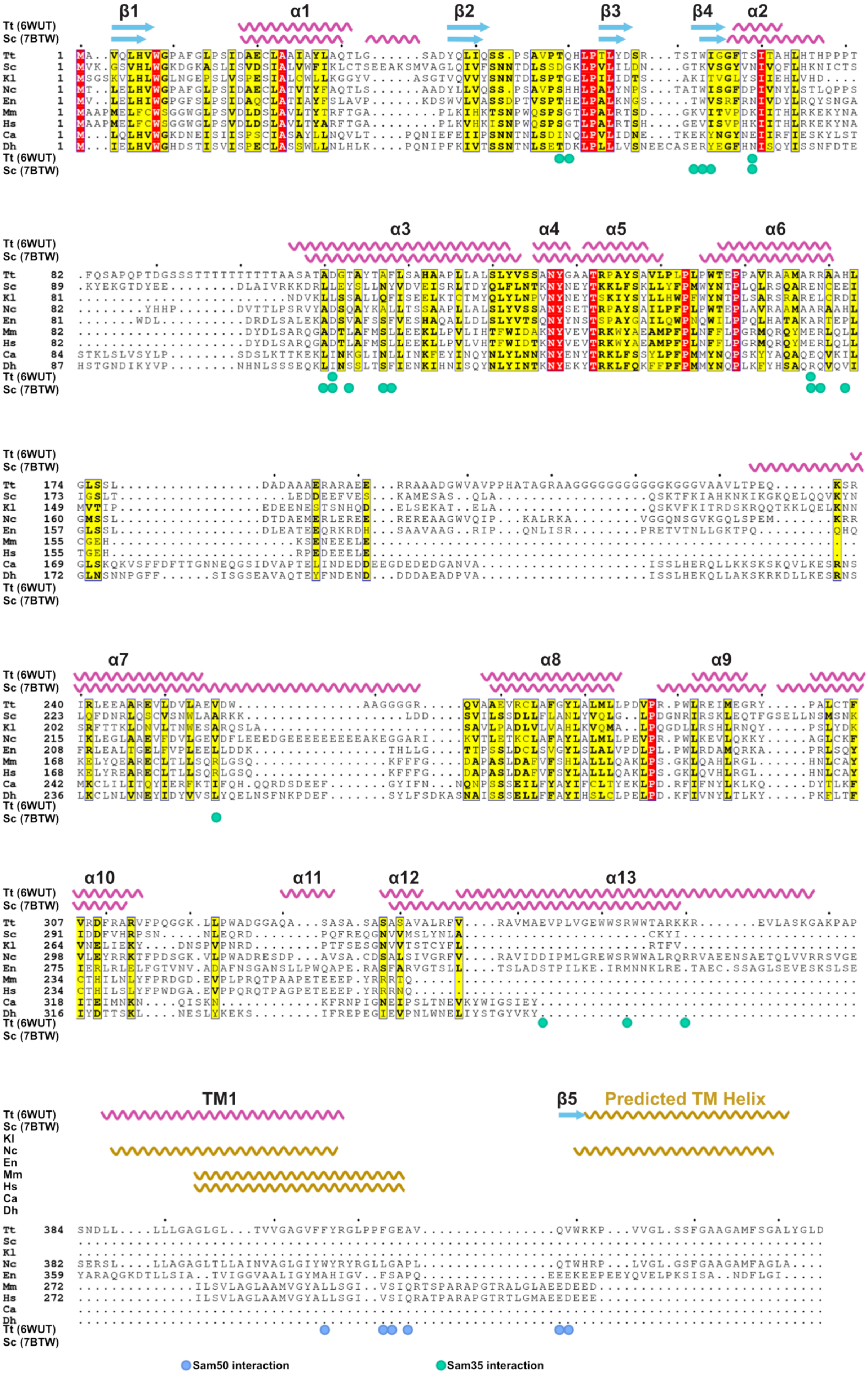

β1
α1
β2
β3
β4
α2
α3
α4
α5
α6
α7
α8
α9
α10
α11
α12
α13
TM1
β5
Predicted TM Helix
Tt (6WUT)
Sc (7BTW)
Tt
Sc
Kl
Nc
En
Mm
Hs
Ca
Dh
Sam50 interaction
Sam35 interaction

Supplementary Figure 3. Sam37 structure-based sequence alignment with predicted transmembrane anchors. Blue arrows represent β-strands, magenta waves represent α-helices. Gold waves represent transmembrane helices predicted by TMHMM 2.0 [65]. Blue circles identify residues that interact with Sam50, green circles identify residues that interact with Sam35. Alignment generated by PROMALS3D [64], adjusted with JalView [62], and colored with ESPript 3.0 [63] based on percent equivalent and 0.6 global score. Tt: *Thermothelomyces thermophilus* (UniProt G2Q6R7; PDB 6WUT), Sc: *Saccharomyces cerevisiae* (UniProt P50110; PDB 7BTW), Kl: *Kluyveromyces lactis* (UniProt Q6CII7), Nc: *Neurospora crassa* (UniProt Q7SFC4), En: *Emericella nidulans* (UniProt Q5B6M7), Mm: *Mus musculus* (UniProt P47802), Hs: *Homo sapiens* isoform 3 (UniProt Q13505-3), Ca: *Candida albicans* (UniProt C4YJG8), Dh: *Debaryomyces hansenii* (UniProt Q6BJD7).

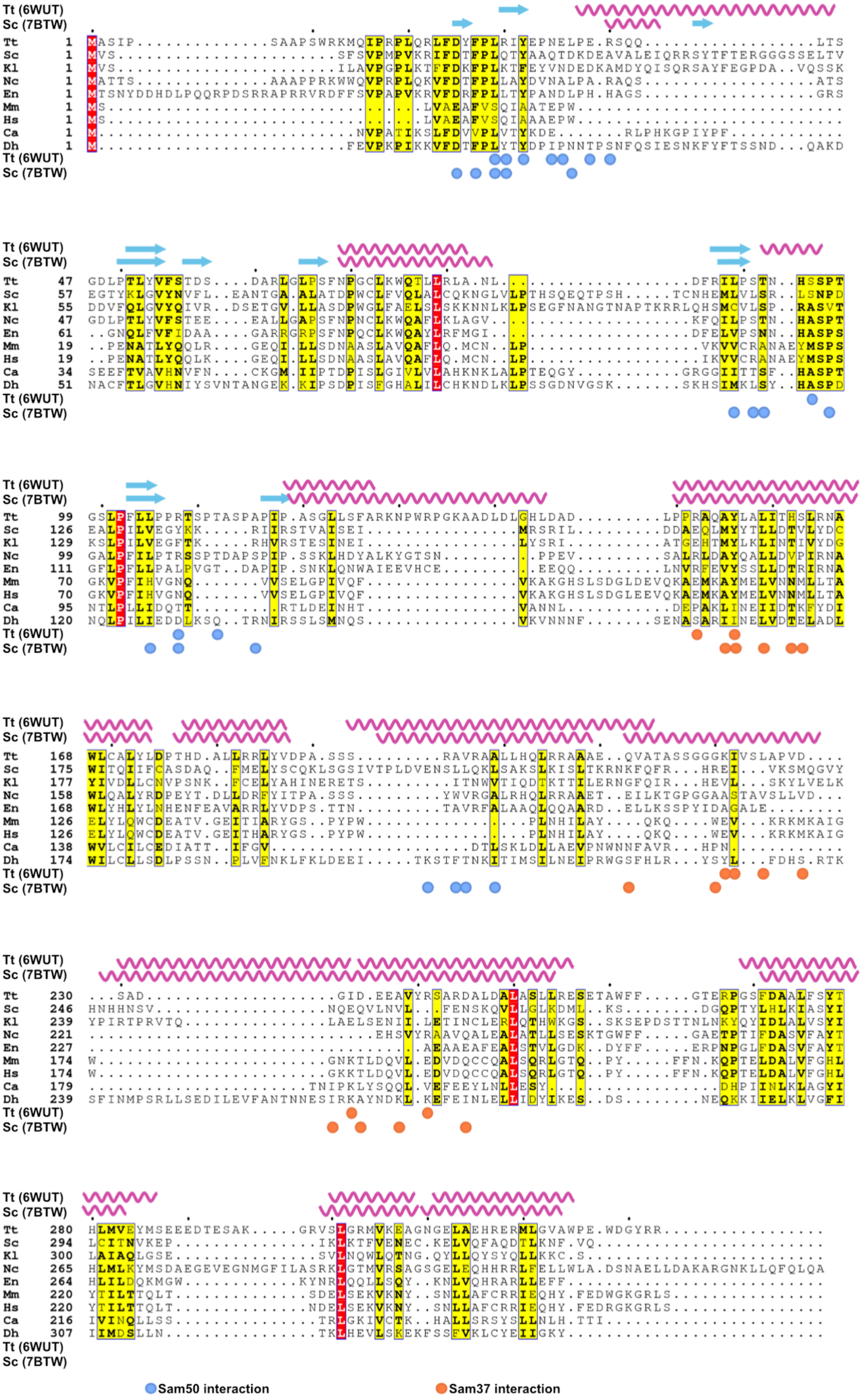
Tt (6WUT)
Sc (7BTW)
Tt
Sc
Kl
Nc
En
Mm
Hs
Ca
Dh
Sam50 interaction
Sam37 interaction

Supplementary Figure 4. Sam35 structure-based sequence alignment. Blue arrows represent β-strands, magenta waves represent α-helices. Blue circles identify residues that interact with Sam50, orange circles identify residues that interact with Sam37. Alignment generated by PROMALS3D [64], adjusted with JalView [62], and colored with ESPript 3.0 [63] based on percent equivalent and 0.6 global score. Tt: *Thermothelomyces thermophilus* (UniProt G2QAT9; PDB 6WUT), Sc: *Saccharomyces cerevisiae* (UniProt P14693; PDB 7BTW), Kl: *Kluyveromyces lactis* (UniProt Q6CMW8), Nc: *Neurospora crassa* (UniProt V5IPB4), En: *Emericella nidulans* (UniProt Q5BFM3), Mm: *Mus musculus* (UniProt O88441), Hs: *Homo sapiens* (UniProt O75431), Ca: *Candida albicans* (UniProt C4YFN0), Dh: *Debaryomyces hansenii* (UniProt Q6BK36).

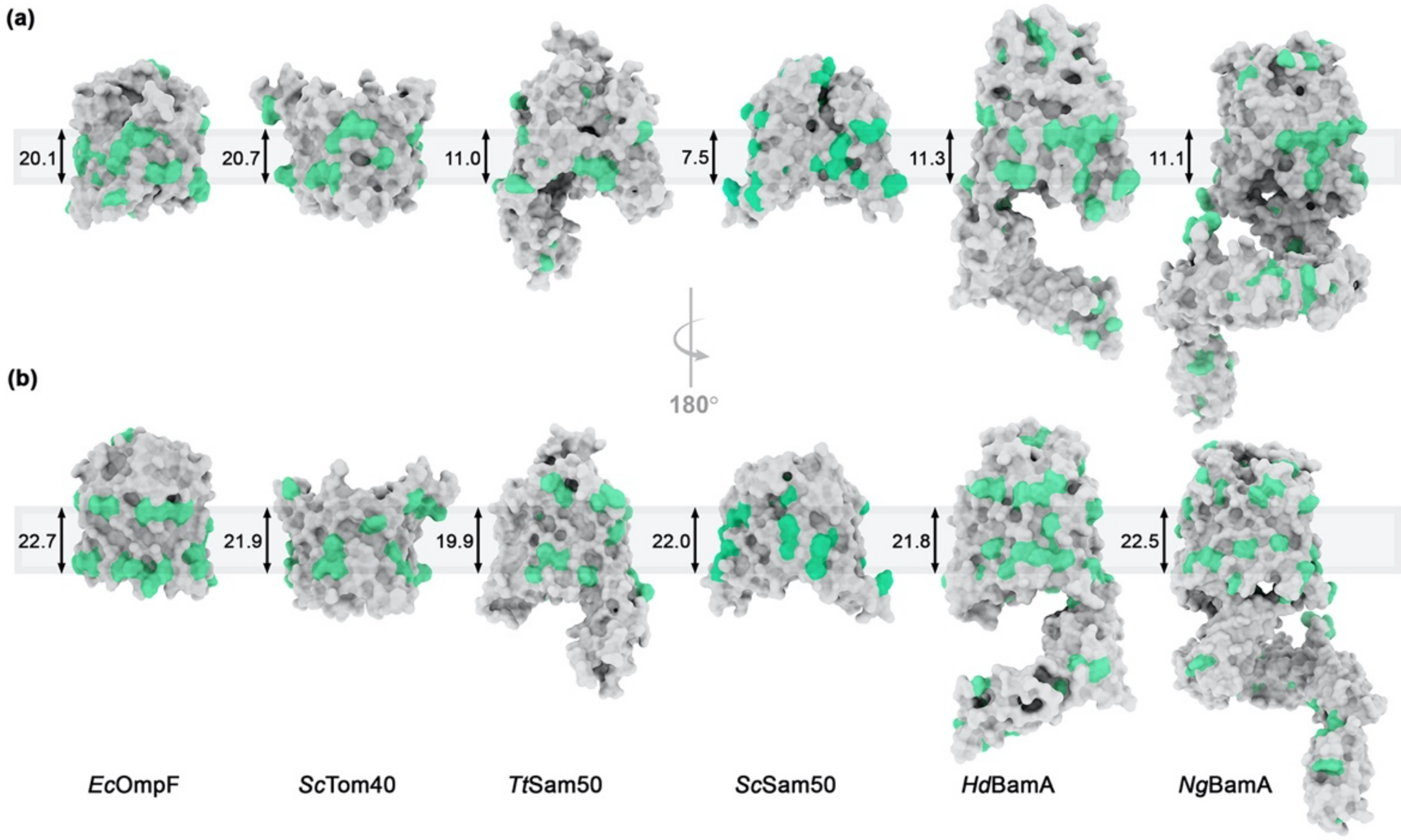

Supplementary Figure 5. Membrane thinning by outer membrane proteins. Comparison of (a) and (b) back views of molecular surfaces of *Ec*OmpF (PDB: 1QJ8), *Sc*Tom40 (6UCU), *Tt*Sam50 (6WUT), *Sc*Sam50 (7BTW), *Hd*BamA (4K3C), and *Ng*BamA (4K3B). Residues that define the membrane boundaries (phenylalanine, tryptophan, tyrosine) are highlighted in green. The arrows indicate the calculated membrane thickness in angstroms (Supplementary Table 3).

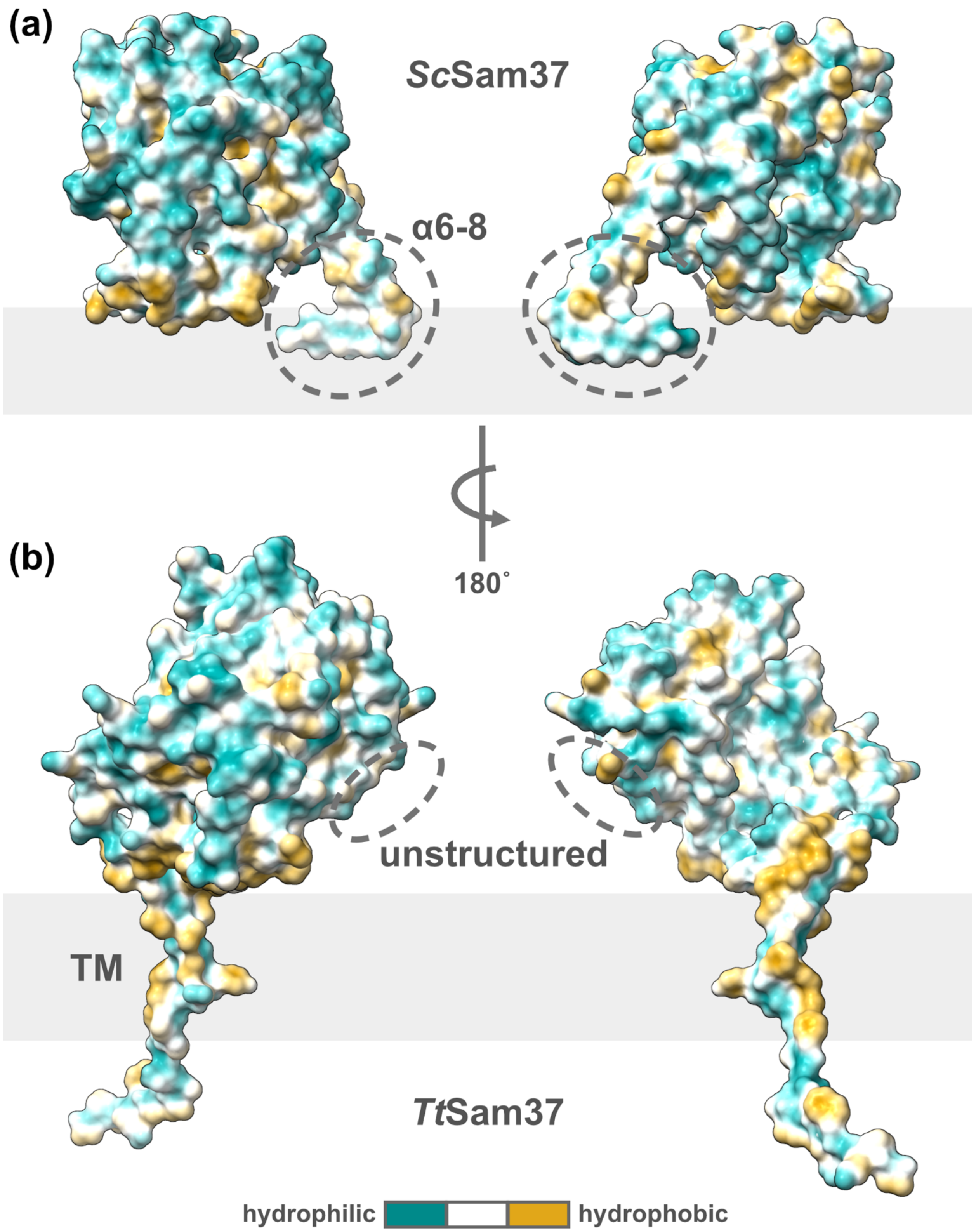

Supplementary Figure 6. Sam37 hydrophobicity. Surface representation with most hydrophilic (dark cyan) and most hydrophobic (dark gold) regions. (a) *Sc*Sam37 from SAM monomer + Mdm10 structure (7BTW) (b) *Tt*Sam37 from SAM monomer structure (6WUT). The dotted circles highlight the position of helices 6-8. Figure generated using ChimeraX molecular lipophilicity potential (mlp) command with default parameters.

Supplementary Table 1: Sam37 transmembrane helix predictions from TMHMM - 2.0 [65].

Species	UniProt ID	Number of TM helices	TM helix Residues
T. thermophilus	G2Q6R7	2	388-410, 423-442
S. cerevisiae	P50110	0	N/A
K. lactis	Q6CII7	0	N/A
N. crassa	Q7SFC4	2	386-408, 421-440
E. nidulans	Q5B6M7	0	N/A
M. musculus	P47802	1	272-294
H. sapiens isoform 3	Q13505-3	1	272-294
C. albicans	C4YJG8	0	N/A
D. hansenii	Q6BJD7	0	N/A

Supplementary Table 2: BamA and Sam50 loop 6 interactions identified in PyMOL (Version 2.4 Schrödinger, LLC) analysis.

T. thermophilus Sam50 (6WUT)			*S. cerevisiae* Sam50 (7BTW)			*N. gonorrhoeae* BamA (4K3B)			*H. ducreyi* BamA (4K3C)		
Loop 6 Residue	Residue 2	Distance (Å)	Loop 6 Residue	Residue 2	Distance (Å)	Loop 6 Residue	Residue 2	Distance (Å)	Loop 6 Residue	Residue 2	Distance (Å)
N372 O	L512 N β16	3.4	S363 OG	A482 O β16	3.4						
N372 ND2	S510 O β16	2.4									
D373 OD2	G369 N L6	2.6	D364 N	G361 O L6	3.5	S658 N	G655 O L6	3.0	S658 N	G655 O L6	2.9
						S658 OG	G654 O L6	2.5	S658 OG	G654 N L6	2.5
									S658 OG	G654 O L6	2.7
D373 OD1	R340 NH1 β11	3.4									
						S658 O	N688 ND2 β12	2.9			
V374 N	G370 O L6	3.5	I365 O	G361 N L6	3.3	V559 N	G655 O L6	3.1	L659 O	G655 N L6	3.1
									L659 N	G655 O L6	2.8
R375 NH2	N402 OD1 β12	3.5				R660 NH2	E692 OE2 β12	2.4	R660 NH1	E698 OE1 β12	3.4
			R366 NH2	N415 OD1 β13	2.6	R660 N	D713 OD2 β13	2.9	R660 N	D721 OD2 β13	3.2
			R366 N	N415 OD1 β13	3.2	R660 NH1	D713 OD2 β13	3.1	R660 NE	D721 OD2 β13	2.8
									R660 NH1	D721 OD1 β13	3.2
			R366 O	S441 OG β14	2.5	R660 O	S751 OG β14	2.8	R660 O	S752 OG β14	2.8
R375 NH1	E484 OE2 β15	3.0	R366 NH1	E456 OE2 β15	3.2						
			R366 NE	E456 OE1 β15	3.0						
G376 O	R500 N L8	3.0	G367 O	R472 N L8	3.8	G661 O	Q782 N L8	3.0	G661 O	E783 N L8	2.7
						G661 N	Q785 OE1 β16	2.4	G661 N	Q786 OE1 β16	2.8
F377 O	Q504 NE2 β16	3.0				Y662 O	Q785 NE2 β16	2.5	F662 O	Q786 NE2 β16	2.8
						Y662 O	G655 N L6	3.5	F662 O	G655 N L6	2.8
						Y662 OH	K774 O L8	3.4			
						Y662 OH	Y750 O β14	3.4			
						E663 OE1	T501 OG1 L3	3.3			
						E663 OE2	T501 OG1 L3	3.2			
									A663 O	S666 NZ L6	3.2
									A663 O	S666 OG L6	2.5
						E663 N	E780 O L8	3.3	A663 N	E781 O L8	2.8
			T370 OG1	D134 OD2 L1	3.6						
									G664 O	K497 NZ L3	2.9

Absolutely conserved (BamA or Sam50 across 10 species)

Semi-conserved (>60% equivalance alignment)

Not conserved

Supplementary Table 3: Membrane thinning measurements from PyMOL (Version 2.4 Schrödinger, LLC).

Structure (PDB ID)	Lateral Gate			Back of Barrel		
	Residue 1	Residue 2	Distance (Å)	Residue 1	Residue 2	Distance (Å)
*Tt*Sam50 (6WUT)	F140	Y154	11.0	F274	Y375	19.9
*Sc*Sam50 (7BTW)	F455	F477	7.5	Y339	F237	22.0
*Ng*BamA (4K3B)	W758	F786	11.1	Y750	F619	22.5
*Hd*BamA (4K3C)	W759	F787	11.3	Y523	Y607	21.8
*Sc*Tom40 (6UCU)	F92	Y114	20.7	F176	Y217	21.9
*Ec*OmpF (2ZFG)	Y4	Y63	20.1	Y182	W214	22.7

Supplementary Movie 1. Flexibility of the Sam50 lateral gate. Conformational changes observed as *Tt*Sam50 (light green) morphs between the closed (dimer 1; PDB 6WUL) and open (dimer 3; 6WUN) conformations. β1-β4 rotate outwards to reach the open conformation which allows room to accommodate precursor protein folding into a new barrel. Sam35 (tan) and Sam37 (light blue) conformations remain unchanged between the two structures. Cryo-EM density of the SAM complex in lipid nanodisc (light blue) defines the extent of the membrane.

Literature Cited for Supplementary Information

[1] E. Bitto, D.B. McKay, Crystallographic Structure of SurA, a Molecular Chaperone that Facilitates Folding of Outer Membrane Porins, Structure. 10 (2002) 1489–1498. https://doi.org/10.1016/S0969-2126(02)00877-8.

[2] S.W. Lazar, R. Kolter, SurA assists the folding of Escherichia coli outer membrane proteins., J. Bacteriol. 178 (1996) 1770–1773. https://doi.org/10.1128/JB.178.6.1770-1773.1996.

[3] T.A. Walton, M.C. Sousa, Crystal Structure of Skp, a Prefoldin-like Chaperone that Protects Soluble and Membrane Proteins from Aggregation, Mol. Cell. 15 (2004) 367–374. https://doi.org/10.1016/j.molcel.2004.07.023.

[4] I.P. Korndörfer, M.K. Dommel, A. Skerra, Structure of the periplasmic chaperone Skp suggests functional similarity with cytosolic chaperones despite differing architecture, Nat. Struct. Mol. Biol. 11 (2004) 1015–1020. https://doi.org/10.1038/nsmb828.

[5] J.G. Sklar, T. Wu, D. Kahne, T.J. Silhavy, Defining the roles of the periplasmic chaperones SurA, Skp, and DegP in Escherichia coli, Genes & Dev. 21 (2007) 2473–2484. https://doi.org/10.1101/gad.1581007.

[6] K.L. Strauch, K. Johnson, J. Beckwith, Characterization of degP, a gene required for proteolysis in the cell envelope and essential for growth of Escherichia coli at high temperature., J. Bacteriol. 171 (1989) 2689–2696. https://doi.org/10.1128/JB.171.5.2689-2696.1989.

[7] C. Spiess, A. Beil, M. Ehrmann, A Temperature-Dependent Switch from Chaperone to Protease in a Widely Conserved Heat Shock Protein, Cell. 97 (1999) 339–347. https://doi.org/10.1016/S0092-8674(00)80743-6.

[8] T. Krojer, M. Garrido-Franco, R. Huber, M. Ehrmann, T. Clausen, Crystal structure of DegP (HtrA) reveals a new protease-chaperone machine, Nature. 416 (2002) 455–459. https://doi.org/10.1038/416455a.

[9] G. Mas, J. Thoma, S. Hiller, The Periplasmic Chaperones Skp and SurA, in: Subcell. Biochem., Springer International Publishing, 2019: pp. 169–186. https://doi.org/10.1007/978-3-030-18768-2_6.

[10] K. Denoncin, J. Schwalm, D. Vertommen, T.J. Silhavy, J.-F. Collet, Dissecting the Escherichia coli periplasmic chaperone network using differential proteomics, Proteomics. 12 (2012) 1391–1401. https://doi.org/10.1002/pmic.201100633.

[11] C.T. Webb, M.A. Gorman, M. Lazarou, M.T. Ryan, J.M. Gulbis, Crystal Structure of the Mitochondrial Chaperone TIM9•10 Reveals a Six-Bladed α-Propeller, Mol. Cell. 21 (2006) 123–133. https://doi.org/10.1016/j.molcel.2005.11.010.

[12] K.N. Beverly, M.R. Sawaya, E. Schmid, C.M. Koehler, The Tim8–Tim13 Complex Has Multiple Substrate Binding Sites and Binds Cooperatively to Tim23, J. Mol. Biol. 382 (2008) 1144–1156. https://doi.org/10.1016/j.jmb.2008.07.069.

[13] M.J. Baker, C.T. Webb, D.A. Stroud, C.S. Palmer, A.E. Frazier, B. Guiard, A. Chacinska, J.M. Gulbis, M.T. Ryan, Structural and Functional Requirements for Activity of the Tim9–Tim10 Complex in Mitochondrial Protein Import, Mol. Biol. Cell. 20 (2009) 769–779. https://doi.org/10.1091/mbc.e08-09-0903.

[14] K. Weinhäupl, C. Lindau, A. Hessel, Y. Wang, C. Schütze, T. Jores, L. Melchionda, B. Schönfisch, H. Kalbacher, B. Bersch, D. Rapaport, M. Brennich, K. Lindorff-Larsen, N. Wiedemann, P. Schanda, Structural Basis of Membrane Protein Chaperoning through the Mitochondrial Intermembrane Space, Cell. 175 (2018) 1365-1379.e25. https://doi.org/10.1016/j.cell.2018.10.039.

[15] I. Sucec, Y. Wang, O. Dakhlaoui, K. Weinhäupl, T. Jores, D. Costa, A. Hessel, M.E. Brennich, D. Rapaport, K. Lindorff-Larsen, B. Bersch, P. Schanda, Structural basis of client specificity in mitochondrial membrane-protein chaperones, BioRxiv. (2020).

[16] S.C. Hoppins, F.E. Nargang, The Tim8-Tim13 Complex of Neurospora crassa Functions in the Assembly of Proteins into Both Mitochondrial Membranes, J. Biol. Chem. 279 (2004) 12396–12405. https://doi.org/10.1074/jbc.M313037200.

[17] N. Wiedemann, K.N. Truscott, S. Pfannschmidt, B. Guiard, C. Meisinger, N. Pfanner, Biogenesis of the Protein Import Channel Tom40 of the Mitochondrial Outer Membrane, J. Biol. Chem. 279 (2004) 18188–18194. https://doi.org/10.1074/jbc.M400050200.

[18] S.A. Paschen, The role of the TIM8-13 complex in the import of Tim23 into mitochondria, EMBO J. 19 (2000) 6392–6400. https://doi.org/10.1093/emboj/19.23.6392.

[19] A.J. Davis, N.N. Alder, R.E. Jensen, A.E. Johnson, The Tim9p/10p and Tim8p/13p Complexes Bind to Specific Sites on Tim23p during Mitochondrial Protein Import, Mol. Biol. Cell. 18 (2007) 475–486. https://doi.org/10.1091/mbc.e06-06-0546.

[20] A.I.C. Höhr, C. Lindau, C. Wirth, J. Qiu, D.A. Stroud, S. Kutik, B. Guiard, C. Hunte, T. Becker, N. Pfanner, N. Wiedemann, Membrane protein insertion through a mitochondrial β-barrel gate, Science (80-.). 359 (2018) eaah6834. https://doi.org/10.1126/science.aah6834.

[21] S.J. Habib, T. Waizenegger, A. Niewienda, S.A. Paschen, W. Neupert, D. Rapaport, The N-terminal domain of Tob55 has a receptor-like function in the biogenesis of mitochondrial β-barrel proteins, J. Cell Biol. 176 (2007) 77–88. https://doi.org/10.1083/jcb.200602050.

[22] M.P. Bos, V. Robert, J. Tommassen, Functioning of outer membrane protein assembly factor Omp85 requires a single POTRA domain, EMBO Rep. 8 (2007) 1149–1154. https://doi.org/10.1038/sj.embor.7401092.

[23] B.M. Burmann, C. Wang, S. Hiller, Conformation and dynamics of the periplasmic membrane-protein–chaperone complexes OmpX–Skp and tOmpA–Skp, Nat. Struct. Mol. Biol. 20 (2013) 1265–1272. https://doi.org/10.1038/nsmb.2677.

[24] S. Reumann, J. Davila-Aponte, K. Keegstra, The evolutionary origin of the protein-translocating channel of chloroplastic envelope membranes: Identification of a cyanobacterial homolog, Proc. Natl. Acad. Sci. 96 (1999) 784–789. https://doi.org/10.1073/pnas.96.2.784.

[25] I. Gentle, K. Gabriel, P. Beech, R. Waller, T. Lithgow, The Omp85 family of proteins is essential for outer membrane biogenesis in mitochondria and bacteria, J. Cell Biol. 164 (2004) 19–24. https://doi.org/10.1083/jcb.200310092.

[26] K. Eckart, L. Eichacker, K. Sohrt, E. Schleiff, L. Heins, J. Soll, A Toc75-like protein import channel is abundant in chloroplasts, EMBO Rep. 3 (2002) 557–562. https://doi.org/10.1093/embo-reports/kvf110.

[27] J. Soll, E. Schleiff, Protein import into chloroplasts, Nat. Rev. Mol. Cell Biol. 5 (2004) 198–208. https://doi.org/10.1038/nrm1333.

[28] P.K. O'Neil, L.G.L. Richardson, Y.D. Paila, G. Piszczek, S. Chakravarthy, N. Noinaj, D. Schnell, The POTRA domains of Toc75 exhibit chaperone-like function to facilitate import into chloroplasts, Proc. Natl. Acad. Sci. 114 (2017) E4868–E4876. https://doi.org/10.1073/pnas.1621179114.

[29] F. Kessler, D. Schnell, Chloroplast biogenesis: diversity and regulation of the protein import apparatus, Curr. Opin. Cell Biol. 21 (2009) 494–500. https://doi.org/10.1016/j.ceb.2009.03.004.

[30] B. Bölter, T. May, J. Soll, A protein import receptor in pea chloroplasts, Toc86, is only a proteolytic fragment of a larger polypeptide, FEBS Lett. 441 (1998) 59–62. https://doi.org/10.1016/S0014-5793(98)01525-7.

[31] L.G. Richardson, M. Jelokhani-Niaraki, M.D. Smith, The acidic domains of the Toc159 chloroplast preprotein receptor family are intrinsically disordered protein domains, BMC Biochem. 10 (2009) 35. https://doi.org/10.1186/1471-2091-10-35.

[32] J. Bauer, K. Chen, A. Hiltbunner, E. Wehrli, M. Eugster, D. Schnell, F. Kessler, The major protein import receptor of plastids is essential for chloroplast biogenesis, Nature. 403 (2000) 203–207.

https://doi.org/10.1038/35003214.

[33] P. Jarvis, An Arabidopsis Mutant Defective in the Plastid General Protein Import Apparatus, Science (80-.). 282 (1998) 100–103. https://doi.org/10.1126/science.282.5386.100.

[34] T. Becker, M. Jelic, A. Vojta, A. Radunz, J. Soll, E. Schleiff, Preprotein recognition by the Toc complex, EMBO J. 23 (2004) 520–530. https://doi.org/10.1038/sj.emboj.7600089.

[35] K. Sohrt, J. Soll, Toc64, a New Component of the Protein Translocon of Chloroplasts, J. Cell Biol. 148 (2000) 1213–1222. https://doi.org/10.1083/jcb.148.6.1213.

[36] M. Sommer, M. Rudolf, B. Tillmann, J. Tripp, M.S. Sommer, E. Schleiff, Toc33 and Toc64-III cooperate in precursor protein import into the chloroplasts of Arabidopsis thaliana, Plant. Cell Environ. 36 (2013) 970–983. https://doi.org/10.1111/pce.12030.

[37] R. Patel, S.C. Hsu, J. Bédard, K. Inoue, P. Jarvis, The Omp85-related chloroplast outer envelope protein OEP80 is essential for viability in Arabidopsis, Plant Physiol. 148 (2008) 235–245. https://doi.org/10.1104/pp.108.122754.

[38] S. Moslavac, O. Mirus, R. Bredemeier, J. Soll, A. Von Haeseler, E. Schleiff, Conserved pore-forming regions in polypeptide- transporting proteins, FEBS J. 272 (2005) 1367–1378. https://doi.org/10.1111/j.1742-4658.2005.04569.x.

[39] M. Töpel, Q. Ling, P. Jarvis, Neofunctionalization within the Omp85 protein superfamily during chloroplast evolution, Plant Signal. Behav. 7 (2012) 161–164. https://doi.org/10.4161/psb.18677.

[40] K. Cline, J. Andrews, B. Mersey, E.H. Newcomb, K. Keegstra, Separation and characterization of inner and outer envelope membranes of pea chloroplasts, Proc. Natl. Acad. Sci. 78 (1981) 3595–3599. https://doi.org/10.1073/pnas.78.6.3595.

[41] K. Inoue, K. Keegstra, A polyglycine stretch is necessary for proper targeting of the protein translocation channel precursor to the outer envelope membrane of chloroplasts, Plant J. 34 (2003) 661–669. https://doi.org/10.1046/j.1365-313X.2003.01755.x.

[42] J. Kim, Y.J. Na, S.J. Park, S.-H. Baek, D.H. Kim, Biogenesis of chloroplast outer envelope membrane proteins, Plant Cell Rep. 38 (2019) 783–792. https://doi.org/10.1007/s00299-019-02381-6.

[43] K.A. Diederichs, X. Ni, S.E. Rollauer, I. Botos, X. Tan, M.S. King, E.R.S. Kunji, J. Jiang, S.K. Buchanan, Structural insight into mitochondrial β-barrel outer membrane protein biogenesis, Nat. Commun. 11 (2020) 3290. https://doi.org/10.1038/s41467-020-17144-1.

[44] S. Gratzer, T. Lithgow, R.E. Bauer, E. Lamping, F. Paltauf, S.D. Kohlwein, V. Haucke, T. Junne, G. Schatz, M. Horst, Mas37p, a novel receptor subunit for protein import into mitochondria, J. Cell Biol. 129 (1995) 25–34. https://doi.org/10.1083/jcb.129.1.25.

[45] D. Milenkovic, V. Kozjak, N. Wiedemann, C. Lohaus, H.E. Meyer, B. Guiard, N. Pfanner, C. Meisinger, Sam35 of the Mitochondrial Protein Sorting and Assembly Machinery Is a Peripheral Outer Membrane Protein Essential for Cell Viability, J. Biol. Chem. 279 (2004) 22781–22785. https://doi.org/10.1074/jbc.C400120200.

[46] N.C. Chan, T. Lithgow, The Peripheral Membrane Subunits of the SAM Complex Function Codependently in Mitochondrial Outer Membrane Biogenesis, Mol. Biol. Cell. 19 (2008) 126–136. http://www.molbiolcell.org/cgi/doi/10.1091/mbc.E07–08–0796.

[47] S.W.K. Lackey, J.G. Wideman, E.K. Kennedy, N.E. Go, F.E. Nargang, The Neurospora crassa TOB Complex: Analysis of the Topology and Function of Tob38 and Tob37, PLoS One. 6 (2011) e25650. https://doi.org/10.1371/journal.pone.0025650.

[48] Y. Guo, Be Cautious with Crystal Structures of Membrane Proteins or Complexes Prepared in Detergents, Crystals. 10 (2020) 86. https://doi.org/10.3390/cryst10020086.

[49] X.-P. Zhang, E. Glaser, Interaction of plant mitochondrial and chloroplast signal peptides with the Hsp70 molecular chaperone, Trends Plant Sci. 7 (2002) 14–21. https://doi.org/10.1016/S1360-1385(01)02180-

X.

[50] A. Matouschek, A. Azem, K. Ratliff, B.S. Glick, K. Schmid, G. Schatz, Active unfolding of precursor proteins during mitochondrial protein import, EMBO J. 16 (1997) 6727–6736. https://doi.org/10.1093/emboj/16.22.6727.

[51] T. Bionda, B. Tillmann, S. Simm, K. Beilstein, M. Ruprecht, E. Schleiff, Chloroplast import signals: The length requirement for translocation in vitro and in vivo, J. Mol. Biol. 402 (2010) 510–523. https://doi.org/10.1016/j.jmb.2010.07.052.

[52] E. Schleiff, J. Soll, Travelling of proteins through membranes: translocation into chloroplasts, Planta. 211 (2000) 449–456. https://doi.org/10.1007/s004250000357.

[53] G. von Heijne, Mitochondrial targeting sequences may form amphiphilic helices., EMBO J. 5 (1986) 1335–1342. https://doi.org/10.1002/j.1460-2075.1986.tb04364.x.

[54] M.A. Razzak, D.W. Lee, Y.-J. Yoo, I. Hwang, Evolution of rubisco complex small subunit transit peptides from algae to plants, Sci. Rep. 7 (2017) 9279. https://doi.org/10.1038/s41598-017-09473-x.

[55] K. Holbrook, C. Subramanian, P. Chotewutmontri, L.E. Reddick, S. Wright, H. Zhang, L. Moncrief, B.D. Bruce, Functional Analysis of Semi-conserved Transit Peptide Motifs and Mechanistic Implications in Precursor Targeting and Recognition, Mol. Plant. 9 (2016) 1286–1301. https://doi.org/10.1016/j.molp.2016.06.004.

[56] C. Ge, E. Spånning, E. Glaser, Å. Wieslander, Import Determinants of Organelle-Specific and Dual Targeting Peptides of Mitochondria and Chloroplasts in Arabidopsis thaliana, Mol. Plant. 7 (2014) 121–136. https://doi.org/10.1093/mp/sst148.

[57] P. Di Tommaso, S. Moretti, I. Xenarios, M. Orobitg, A. Montanyola, J.M. Chang, J.F. Taly, C. Notredame, T-Coffee: A web server for the multiple sequence alignment of protein and RNA sequences using structural information and homology extension, Nucleic Acids Res. 39 (2011) 13–17. https://doi.org/10.1093/nar/gkr245.

[58] F. Armougom, S. Moretti, O. Poirot, S. Audic, P. Dumas, B. Schaeli, V. Keduas, C. Notredame, Expresso: Automatic incorporation of structural information in multiple sequence alignments using 3D-Coffee, Nucleic Acids Res. 34 (2006) 604–608. https://doi.org/10.1093/nar/gkl092.

[59] O. O'Sullivan, K. Suhre, C. Abergel, D.G. Higgins, C. Notredame, 3DCoffee: Combining Protein Sequences and Structures within Multiple Sequence Alignments, J. Mol. Biol. 340 (2004) 385–395. https://doi.org/10.1016/j.jmb.2004.04.058.

[60] O. Poirot, K. Suhre, C. Abergel, E. O'Toole, C. Notredame, 3DCoffeeıgs: A web server for combining sequences and structures into a multiple sequence alignment, Nucleic Acids Res. 32 (2004) 37–40. https://doi.org/10.1093/nar/gkh382.

[61] C. Notredame, D.G. Higgins, J. Heringa, T-coffee: A novel method for fast and accurate multiple sequence alignment, J. Mol. Biol. 302 (2000) 205–217. https://doi.org/10.1006/jmbi.2000.4042.

[62] A.M. Waterhouse, J.B. Procter, D.M.A. Martin, M. Clamp, G.J. Barton, Jalview Version 2-A multiple sequence alignment editor and analysis workbench, Bioinformatics. 25 (2009) 1189–1191. https://doi.org/10.1093/bioinformatics/btp033.

[63] X. Robert, P. Gouet, Deciphering key features in protein structures with the new ENDscript server, Nucleic Acids Res. 42 (2014) 320–324. https://doi.org/10.1093/nar/gku316.

[64] J. Pei, B.-H. Kim, N. V. Grishin, PROMALS3D: a tool for multiple protein sequence and structure alignments, Nucleic Acids Res. 36 (2008) 2295–2300. https://doi.org/10.1093/nar/gkn072.

[65] A. Krogh, B. Larsson, G. von Heijne, E.L.L. Sonnhammer, Predicting transmembrane protein topology with a hidden markov model: application to complete genomes, J. Mol. Biol. 305 (2001) 567–580. https://doi.org/10.1006/jmbi.2000.4315.
